# ScGeo reveals non-canonical trajectories beyond RNA velocity in radiation-induced hematopoietic recovery

**DOI:** 10.1093/bioadv/vbag266

**Published:** 2026-09-09

**Authors:** Yu-Chen Liu, Kengo Yoshida

**Affiliations:** Radiation Effects Research Foundation (RERF), Hiroshima, 5-2 Hijiyama Park, Minami-ku, 732-0815, Japan; Laboratory for Human Immunology (Single Cell Genomics), WPI Immunology Frontier Research Center (IFReC), Osaka University, Osaka, 565-0871, Japan; Radiation Effects Research Foundation (RERF), Hiroshima, 5-2 Hijiyama Park, Minami-ku, 732-0815, Japan

## Abstract

Low-dimensional representations are central to single-cell RNA sequencing analysis, yet perturbation-associated geometry is often interpreted visually without explicit assessment of estimator, sampling, or representation dependence. We introduce ScGeo, a representation-aware framework that treats embeddings as quantitative objects and reports robust state displacement, biological-sample uncertainty, local geometric preservation, cross-representation stability, distributional change, and agreement between condition-dependent displacement and independently supplied dynamics estimates. In GSE280305 post-irradiation hematopoietic recovery, ScGeo identified heterogeneous D8-to-D21 cluster displacement and partial agreement between geometric shifts and RNA velocity, while avoiding interpretation of time-point mixing as proof of valid integration. A prespecified synthetic benchmark showed that robust center estimators reduced outlier sensitivity, global representation corruption was detectable, and fine-grained localization of local distortion remained limited. A GSE132188-derived pancreatic-development workflow provided descriptive geometry-dynamics validation. In GSE249479, inflammatory effects in hematopoietic stem and progenitor cells were broadly stable across the primary representation ensemble but remained descriptive because biological-replicate identity was unavailable. In replicate-aware GSE211713 lung-radiation analysis, early 17 Gy effects were representation-sensitive, whereas late remodeling was stable in five of six major compartments. ScGeo provides an auditable downstream layer for distinguishing stable, neutral, insufficient-coverage, and representation-sensitive interpretations rather than assuming any single latent space is biologically definitive.

## 1 Introduction

Single-cell RNA sequencing (scRNA-seq) enables high-resolution analysis of transcriptional heterogeneity across biological conditions and time (Reinius *et al.* 2016). Most workflows construct quantitative representations, including principal components and nonlinear manifolds, for clustering, visualization, integration, and trajectory analysis (Wolf *et al.* 2018). Although distances, gradients, and cluster separations in these representations are routinely interpreted, the stability of perturbation-associated geometric conclusions is seldom audited across estimators, biological samples, local neighborhood structure, and alternative representations.

Embedding-based representations have long been used in single-cell analysis, including PCA-based dimensionality reduction, clustering, and trajectory inference. In representation learning, distances, directions, and distributional structure are treated as measurable properties of a learned coordinate system (Bengio *et al.* 2013, Yao and Zheng 2023). This perspective motivates quantitative analysis of single-cell representations while recognizing that such geometry is conditional on how the representation was constructed.

Batch correction and data integration further complicate the interpretation of embedding geometry. Methods such as Scanorama (Hie *et al.* 2019) aim to harmonize samples across technical or biological batches while preserving meaningful structure. However, evaluation of integration performance commonly relies on global mixing metrics or visual inspection, which may fail to capture structured biological transitions embedded within the manifold. In perturbation studies—such as irradiation, infection, or drug exposure—cellular responses are expected to induce directional remodeling of cellular state space over time. Batch correction and representation learning further complicate the interpretation of embedding geometry because distances and neighborhood relationships can change with the integration method, latent model, dimensionality, and random initialization. No integration method can be assumed to provide a universally faithful biological coordinate system, and biological-condition mixing is not necessarily a desirable objective when the condition itself is the signal of interest. Consequently, perturbation-associated conclusions should be evaluated across prespecified quantitative representations and accompanied by diagnostics of neighborhood preservation, local distortion, and conclusion stability.

Here, we introduce ScGeo, a representation-aware framework for quantifying perturbation-associated geometry in single-cell embeddings. ScGeo does not learn a new latent representation, infer pseudotime, estimate an optimal-transport coupling, or replace RNA-velocity and fate-inference methods. Instead, it treats existing quantitative representations as objects whose perturbation-associated structure can be measured, compared, and audited.

ScGeo combines several complementary evidence layers: robust condition-dependent displacement of annotated cellular states, uncertainty across independent biological samples, stability across prespecified representations, local-neighborhood preservation and distortion, distributional change, and agreement between observed geometric displacement and an independently supplied dynamics estimate. The individual quantities used in these layers draw on established geometric and statistical principles. The framework-level contribution lies in connecting them through a common representation-aware workflow that explicitly distinguishes stable effects, stable neutral findings, insufficient coverage, and representation-sensitive conclusions.

This design complements rather than replaces existing methodological classes. Manifold-learning and integration methods construct representations; optimal-transport and perturbation-prediction methods estimate mappings or counterfactual states; trajectory-inference methods reconstruct cellular progressions; and RNA velocity or CellRank estimate directional dynamics. ScGeo instead asks whether an observed perturbation-associated geometric interpretation is robust to the estimator, biological sampling, local representation structure, and the choice of latent space, and whether that interpretation agrees with available dynamical evidence.

Recent single-cell representation-learning approaches increasingly incorporate pretrained foundation models and deep latent architectures (Ergen *et al.* 2025). For example, scPASI combines scFoundation-derived embeddings with residual variational-autoencoder refinement and phenotype-guided ensemble regression to identify phenotype-associated cellular subpopulations (Zhao *et al.* 2026). Such methods construct or refine task-oriented representations, whereas ScGeo operates downstream of representation learning and evaluates the robustness and geometric interpretation of perturbation-associated differences within supplied coordinate systems.

ScGeo is therefore representation-aware rather than integration-specific. It can analyze uncorrected PCA coordinates, corrected embeddings, variational latent spaces, diffusion representations, or other quantitative coordinates, provided that their biological and technical interpretation is specified. Scanorama is used in the original hematopoietic case study, but it is not required by the framework and is not treated as a uniquely valid representation.

In parallel, dynamical modeling approaches including RNA velocity (La Manno *et al.* 2018, Bergen *et al.* 2020, 2021) and probabilistic fate inference (e.g. CellRank) (Lange *et al.* 2022) have enabled reconstruction of directional progression and terminal states from single-cell transcriptomic data. These methods estimate vector fields and lineage probabilities that describe cellular trajectories. However, their relationship to embedding-level geometric structure remains largely implicit. It is unclear whether condition-dependent shifts in embedding space are consistent with inferred dynamical flow, or whether additional structure exists beyond transcriptional kinetics.

We demonstrate ScGeo using radiation-induced hematopoietic recovery in GSE280305 as the principal biological case study and evaluate the framework through complementary controlled and public-data analyses. First, prespecified synthetic benchmarks test estimator robustness, biological-sample uncertainty, representation corruption, local geometric distortion, and geometry–dynamics agreement under known conditions. Second, a public murine pancreatic endocrinogenesis dataset derived from GSE132188 (Bastidas-Ponce *et al.* 2019) evaluates geometry–dynamics relationships in a distinct developmental system. Third, GSE249479 (Zeng *et al.* 2026), a public human HSPC inflammatory xenograft dataset, evaluates descriptive perturbation geometry across multiple representations and comparison with official R Augur analysis. Finally, GSE211713 (Curras-Alonso *et al.* 2023), a public mouse-lung radiation dataset containing 20 independent mouse libraries, evaluates replicate-aware geometric remodeling across early and late radiation responses.

These analyses retain radiation-induced hematopoietic recovery as the central biological motivation while testing whether ScGeo can operate across different tissues, perturbations, representation types, and experimental designs. Because the available replication and study design differ among datasets, each validation setting is assigned an explicit inference scope rather than being interpreted as equivalent evidence.

## 2 Materials and methods

**Figure 1:**
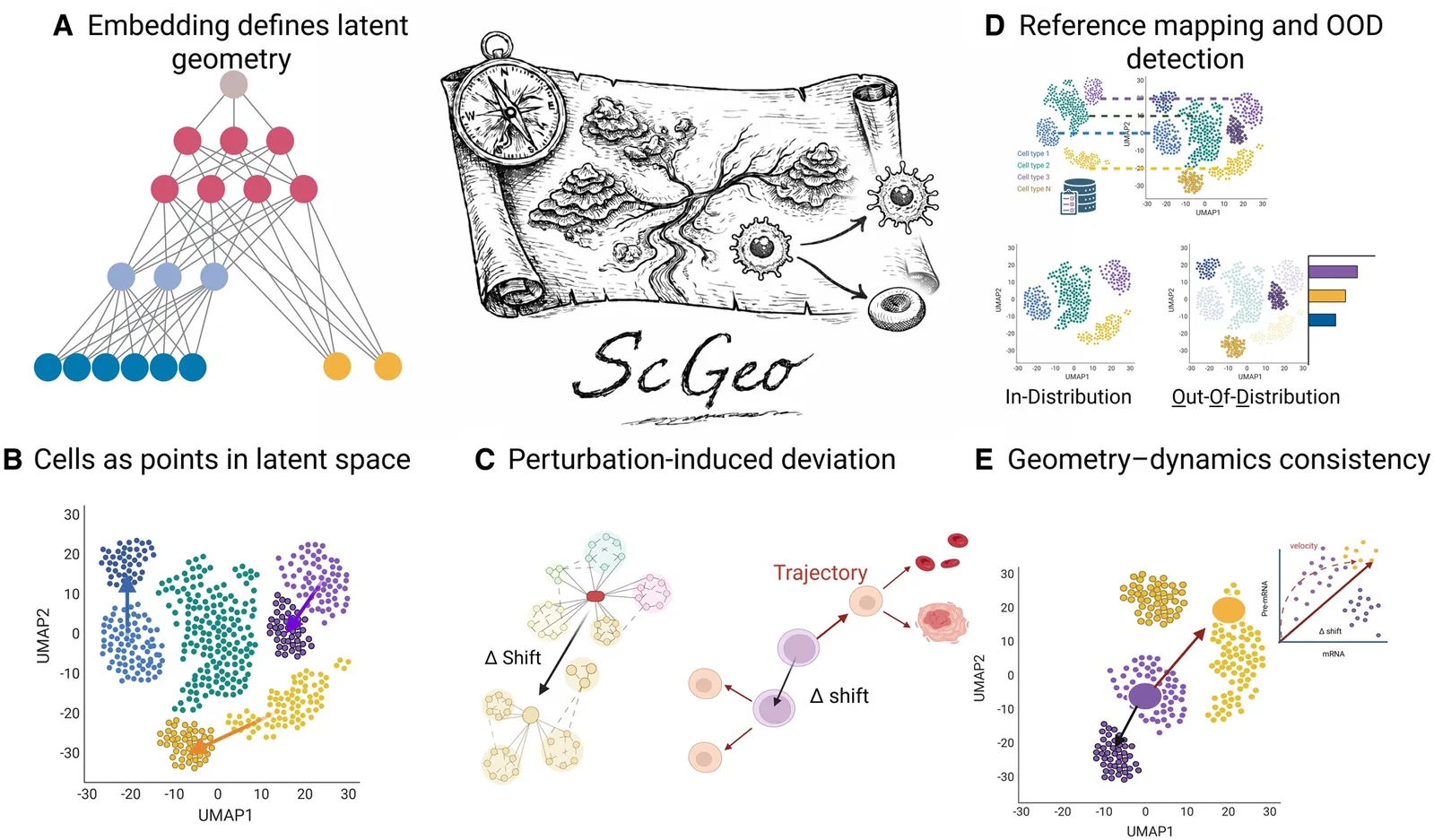
Conceptual overview of ScGeo: embedding geometry as a quantitative representation of cellular state space. (A) Schematic of latent embedding structure. High-dimensional gene expression profiles are projected into a low-dimensional space, where cells form structured manifolds. (B) Cells represented as points in latent space. Distinct cell populations occupy separable regions, and trajectories correspond to directional transitions within the embedding. (C) Perturbation-induced geometric deviation. External perturbations (e.g. irradiation) induce displacement of cell populations in embedding space, quantified as geometric shift (Δ). (D) Reference mapping and out-of-distribution (OOD) detection. Cells are evaluated relative to a reference manifold, enabling identification of in-distribution and OOD states. (E) Geometry–dynamics consistency. Alignment between geometric displacement vectors and RNA velocity vectors provides a quantitative measure of concordance between embedding-level transitions and inferred transcriptional dynamics. Created in BioRender. Liu, Y. (2026) https://BioRender.com/99l0f4m

Figure 1 summarizes the conceptual design of ScGeo. The framework treats one or more supplied quantitative representations as coordinate systems in which perturbation-associated structure can be measured and audited. Its evidence layers include local neighborhood structure, condition-dependent state displacement, distributional divergence, reference-deviation analysis, cross-representation stability, and comparison with independently supplied dynamics estimates. The GSE280305 case study uses a Scanorama-derived representation for displacement and geometry–dynamics analyses. Quantitative representation comparisons in the validation analyses use PCA, scVI, and diffusion representations, with UMAP (McInnes *et al*. 2018) reserved for visualization.

### 2.1 Dataset and preprocessing

**Figure 2:**
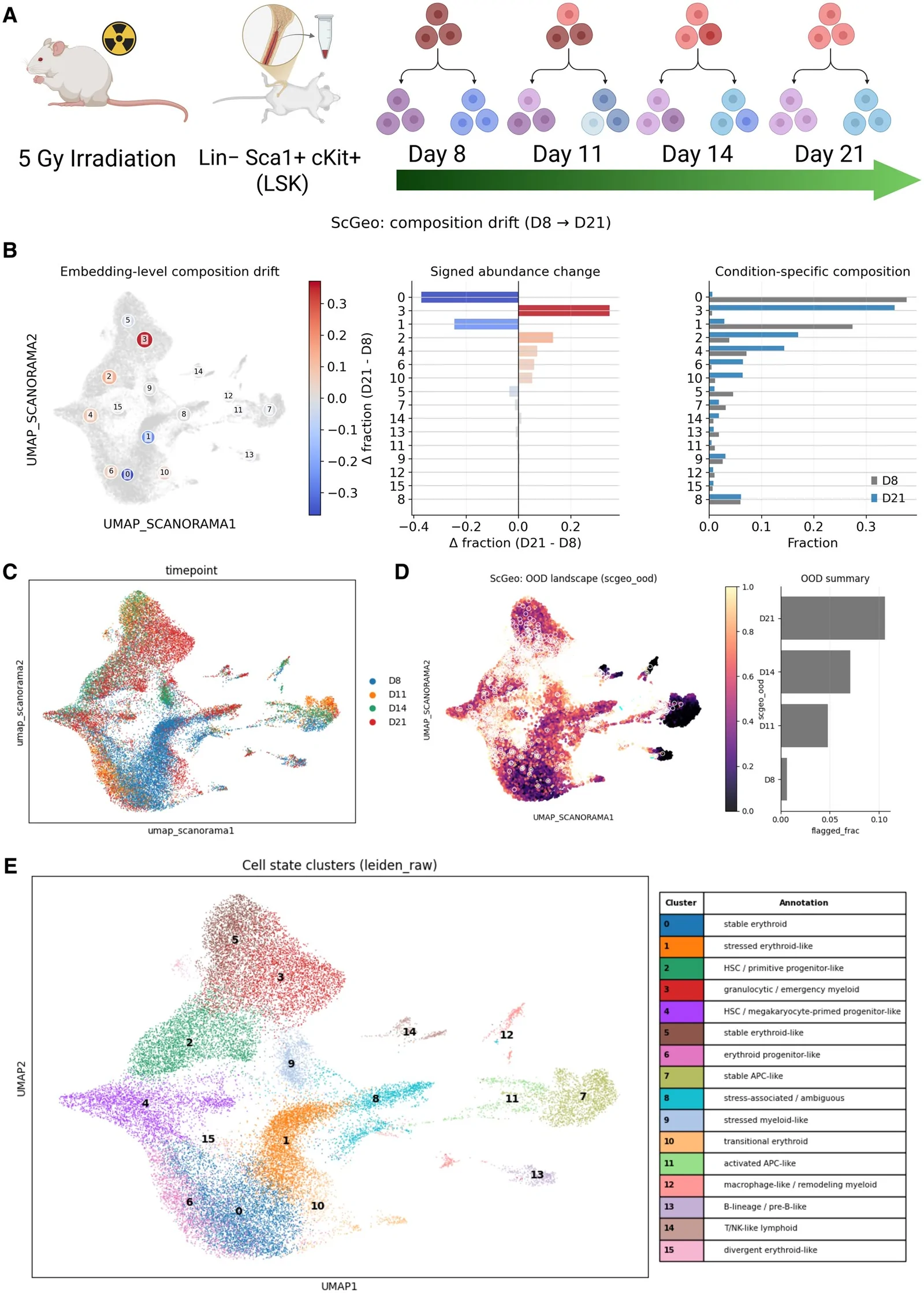
ScGeo maps post-irradiation redistribution of hematopoietic state space relative to D8. (A) Experimental design. C57BL/6J mice were exposed to 5 Gy total body irradiation. Lin⁻ Sca1⁺ cKit⁺ (LSK) hematopoietic stem/progenitor cells were profiled at days 8, 11, 14, and 21 post-irradiation. (B) Embedding-level composition drift (D8 → D21). Left: spatial map of cluster-wise fractional change across the embedding. Middle: signed abundance change for each cluster. Right: timepoint-specific cluster fractions at D8 and D21. (C) Scanorama UMAP colored by sampling time point. The panel visualizes the distribution of D8, D11, D14, and D21 cells and is not used as evidence that integration preserved a uniquely valid biological geometry. (D) D8-reference deviation landscape. Left: per-cell reference-deviation scores projected onto the embedding, calculated relative to the D8 cell distribution. Right: fraction of cells exceeding the fixed reference-deviation threshold at each time point. D8 is the earliest sampled post-irradiation condition and is not an untreated baseline. (E) Cell state clustering and annotation. UMAP colored by Leiden clusters with corresponding biological annotations, including erythroid, myeloid/APC, progenitor-like, and stress-associated populations.

We analyzed the public scRNA-seq dataset GSE280305, comprising Lin⁻ Sca1⁺ cKit⁺ (LSK) hematopoietic stem/progenitor cells collected at days 8, 11, 14, and 21 after 5 Gy irradiation (Fig. 2A). Because the public study design did not provide an untreated reference within this analysis, comparisons among time points are interpreted as post-irradiation differences rather than recovery toward a healthy baseline.

Raw count matrices were processed using standard preprocessing procedures, including quality control filtering, library size normalization, logarithmic transformation, highly variable gene (HVG) selection, and principal component analysis (PCA).

Let $X\in R^{n\times p}$ denote the normalized gene expression matrix, where n is the number of cells and p is the number of selected HVGs. Dimensionality reduction was performed via PCA:


Z=XW

where $W\in R^{p\times }$contains the first d principal component loadings, and $Z\in R^{n\times d}\;$is the low-dimensional embedding used for downstream geometric analyses. Batch correction and integration were performed using Scanorama (Hie *et al.* 2019), yielding the integrated embedding: $Z_{\mathrm{int}}\in R^{n\times d}$.

### 2.2 Validation datasets and inference scopes

ScGeo was evaluated using one controlled benchmark and three public validation workflows in addition to the original GSE280305 radiation-recovery analysis. The validation settings were selected to test complementary aspects of the framework and were not treated as supporting interchangeable levels of biological inference.

Synthetic benchmark. Controlled simulations were used to evaluate estimator robustness, biological-sample uncertainty, representation corruption, local geometric distortion, and geometry–dynamics agreement under known truth. Calibration and held-out evaluation seeds were separated before final assessment, and the prespecified thresholds were not retuned after evaluation.

### 2.3 Public pancreatic-development workflow (GSE132188-derived)

A public pancreatic-development dataset with available scVelo and CellRank outputs was used to evaluate agreement between perturbation geometry and developmental dynamics across quantitative representations. This analysis was treated as descriptive validation because the dynamics estimates arise from related transcriptional-transition models rather than from an independent experimental ground truth.

GSE249479. This public human HSPC inflammatory xenograft dataset was used to evaluate descriptive TNF- and LPS-associated geometry across PCA, diffusion, and scVI representations. No valid cell-level biological-replicate identity could be recovered from the available object. All conclusions from this workflow were therefore labeled descriptive. Official R Augur (Skinnider *et al.* 2021) analysis was used as the principal external comparator; the retained Python approximation was treated only as implementation sensitivity.

GSE211713. This public whole-lung radiation dataset comprised 20 independent mouse libraries spanning control, 10 Gy, and 17 Gy conditions across multiple post-exposure months. Prespecified primary contrasts compared control with early 17 Gy, control with late 17 Gy, and early with late 17 Gy groups. Mouse-level state centers, biological-sample bootstrap resampling, and exact label permutations were used. Because different mice were sampled at each time point, all temporal comparisons were interpreted as cross-sectional rather than longitudinal.

Detailed preprocessing, representation construction, analysis parameters, and inference scopes for the validation workflows are provided in Supplementary Methods S2–S12 and Table S1.

### 2.4 RNA velocity and fate inference

RNA velocity was computed using scVelo (Bergen *et al.* 2020), resulting in per-cell velocity vectors $v_{i}\in R^{d},i=1,\ldots ,n$. We denote the set of velocity vectors as $V=\{v_{1},\ldots ,v_{n}\}$.

Cell fate inference was performed using CellRank with GPCCA-based macrostate decomposition (Lange *et al.* 2022). For each cell i, fate probabilities are defined as:


$$f_{i}=(f_{i1},\ldots ,f_{ik}),\sum_{j=1}^{k}f_{ij}=1$$

where k is the number of macrostates.

### 2.5 ScGeo framework

ScGeo treats a low-dimensional embedding


$$Z=\{z_{1},\ldots ,z_{n}\},z_{i}\in R^{d}$$

as a geometric representation of cellular state space. All computed quantities are stored in adata.uns[scgeo].

### 2.6 Robust and sample-aware geometric displacement

For a cellular state g, condition c, and quantitative representation Z, let $Z_{g,c}$denote the cells assigned to that state and condition. ScGeo defines a state center using a prespecified estimator T:


$${\overset{\prime }{\mu }}_{g,c}=T(Z_{g,c}),$$

where Tmay be the arithmetic mean, coordinate-wise median, trimmed mean, or geometric median. The condition-dependent displacement vector is


$$I_{0}=\{i:c_{i}=0\},I_{1}=\{i:c_{i}=1\}\Delta_{g}={\overset{\prime }{\mu }}_{g,1}-{\overset{\prime }{\mu }}_{g,0},$$

with magnitude $∥\Delta_{g}{∥}_{2}$. The arithmetic mean provides a transparent estimate of expected location but is sensitive to outliers. Median-, trimmed-mean-, and geometric-median estimators provide alternative robustness properties and are evaluated through estimator-sensitivity analysis rather than assumed to be universally superior.

When independent biological samples are available, state centers are first computed separately for each biological sample. Condition-level displacement and uncertainty are then estimated from the distribution of sample-level centers. Bootstrap resampling is performed over biological samples, not over individual cells. The resulting outputs include displacement magnitude, direction, interval estimates, sample coverage, normalized effect size, and direction or sign stability.

Analyses without a recoverable biological-sample identifier are explicitly labeled descriptive and do not receive biological-sample uncertainty estimates.

Arithmetic-mean displacement is implemented in scgeo.tl.shift(). Robust and biological-sample-aware displacement, including coordinate-wise median, trimmed mean, and geometric median estimators, is implemented in scgeo.tl.robust_shift(). The behavior of the alternative center estimators under outlier contamination and biological-sample heterogeneity is evaluated in Fig. S1 and Table S2.


$$\Delta =\mu_{1}-\mu_{0}$$

$${\left\|\Delta \right\|}_{2}=\sqrt{\Delta^{T}\Delta }$$

$$\Delta_{g}=\mu_{1,g}-\mu_{0,g}$$

### 2.7 Local mixing score (Mixscore)

Let $N_{k}(i)$denote the set of k-nearest neighbors of cell i, and let b denote a categorical label, such as batch or experimental condition, depending on the analysis context. In this study, we primarily use condition labels (e.g. time points), while the formulation is general and can also be applied to batch labels for assessing integration quality.

The local mixing score is defined as:


$${\mathrm{mixscore}}_{i}=\frac{1}{k}\sum_{j\in N_{k}(i)}1(b_{j}\neq b_{i}),$$

where 1(⋅) is the indicator function. This formulation is equivalent to a local heterogeneity measure and provides a computationally efficient proxy for neighborhood entropy-based mixing metrics.

This score quantifies the fraction of neighbors whose labels differ from that of the focal cell. UMAP-derived mixscores are not used as quantitative evidence for integration quality or representation robustness in this study; quantitative representation comparisons are performed directly in the prespecified PCA, scVI, and diffusion spaces.

Implemented in: scgeo.tl.mixscore().

### 2.8 Distributional divergence test

To quantify distributional differences between two cell sets A and B, we use the energy distance:


$$\begin{array}{c} E(A,B)=\frac{2}{\left|A||B\right|}\sum_{i\in A}\sum_{j\in B}\left\|z_{i}-z_{j}\right\|\\ -\frac{1}{{\left|A\right|}^{2}}\sum_{i,i^{\prime }\in A}\left\|z_{i}-z_{i^{\prime }}\right\|\\ -\frac{1}{{\left|B\right|}^{2}}\sum_{j,j^{\prime }\in B}\left\|z_{j}-z_{j^{\prime }}\right\| \end{array}$$

Energy distance was chosen for its non-parametric nature and its sensitivity to both location and distributional differences in high-dimensional embeddings.

Statistical significance was assessed by permutation testing. Let E_obs denote the energy distance calculated from the observed labels. The labels were permuted R times while preserving group sizes, and the statistic was recalculated for each permutation r. The permutation *P*-value was:


$$p=\frac{1+\sum_{r=1}^{R}1(E^{\left(r\right)}\geq E_{\mathrm{obs}})}{R+1},$$

where R is the total number of permutations and the indicator equals 1 when the permuted statistic is at least as large as E_obs. Adding 1 to the numerator and denominator prevents a zero estimated *P*-value under finite permutation sampling. The permutation unit must match the supported inference scope: biological samples when valid sample identity is available, or otherwise explicitly labeled cell-level computational sensitivity.

Implemented in: scgeo.tl.distribution_test().

### 2.9 Alternative distributional metrics

In addition to energy distance, ScGeo supports alternative measures of distributional divergence in embedding space. These include density-based overlap metrics such as the Bhattacharyya coefficient and Hellinger distance (Devroye *et al.* 1996), estimated using k-nearest neighbor density approximations, as well as sliced Wasserstein distance (Bonneel *et al.* 2015) computed via random projections. These metrics are included for extensibility of the framework but were not required to support the conclusions of this study.

### 2.10 Velocity–displacement alignment

For a subset or cluster g, the mean RNA velocity is:


$$\bar{v_{g}}=\frac{1}{\left|I_{g}\right|}\sum_{i\in I_{g}}v_{i}$$

Alignment between geometric displacement and velocity is quantified using cosine similarity:


$$\text{cos}(\theta_{g})=\frac{\Delta_{g}^{T}\bar{v_{g}}}{{\left\|\Delta_{g}\right\|}_{2}{\left\|\bar{v_{g}}\right\|}_{2}}$$

Implemented in: scgeo.tl.velocity_shift_alignment().

### 2.11 Reference deviation analysis

ScGeo can quantify deviation from a prespecified reference distribution in a supplied representation. The resulting score is conditional on the chosen reference, coordinate system, neighborhood definition, and threshold and should not be interpreted as a universal out-of-distribution probability.

The reference-deviation implementation operates on coordinates stored in an AnnData object and returns higher values for cells lying farther from the prespecified reference distribution. Dataset-specific reference choice and thresholding are reported with each application.

### 2.12 GSE280305 D8-reference deviation score

For GSE280305, D8—the earliest sampled post-irradiation time point—was designated as the reference condition. Cells from D11, D14, and D21 were evaluated relative to the D8 distribution in the analyzed Scanorama-derived representation. The score therefore measures departure from the D8 post-irradiation reference, not deviation from an untreated or healthy state. The reference set, representation, neighborhood parameters, and threshold were held fixed across time points.

### 2.13 Differential expression and pathway enrichment analysis

Differential expression analysis between canonical and discordant cell populations was performed using Scanpy (Wolf *et al.* 2018), with genes ranked based on log-fold change and statistical significance.

Pathway enrichment analysis was conducted using over-representation analysis (ORA) implemented in the gseapy Python package (Fang *et al.* 2023). Significantly upregulated genes from each comparison were tested against curated gene sets, including Gene Ontology (GO) Biological Process (The Gene Ontology Consortium 2015) and MSigDB Hallmark pathways (Castanza *et al.* 2023). Enrichment significance was evaluated using a hypergeometric test, and resulting *P*-values were adjusted for multiple testing using the Benjamini–Hochberg procedure (Virtanen *et al.* 2020). Pathways with adjusted *P*-value < 0.05 were considered statistically significant and were ranked by adjusted *P*-value.

### 2.14 Representation ensemble and robustness assessment

To evaluate whether ScGeo conclusions depended on a particular latent representation, we prespecified primary and sensitivity representations for each validation workflow. PCA30, PCA50, and a 20-dimensional scVI latent representation were used as the primary ensemble where available. PCA20 was retained as a dimensionality-sensitivity view derived from the shared PCA basis, and diffusion maps were analyzed as an exploratory sensitivity representation. Related PCA views were not counted as independent confirmations. UMAP coordinates were used for visualization only and were excluded from quantitative displacement and distributional analyses.

For every eligible state and contrast, ScGeo was evaluated separately in each representation. Representation agreement was summarized using state-effect rank concordance, directional agreement, and the stability of the resulting evidence classification. Leave-one-primary-representation-out analyses assessed whether a conclusion depended on any single primary representation.

Local geometric stability was evaluated separately from state displacement. Diagnostics included neighborhood overlap, edge retention, graph connectivity, and normalized local distortion relative to a prespecified reference representation. These quantities were used as sensitivity diagnostics and were not combined with displacement into a composite score.

Integration correction was applied only when a documented technical batch factor could be distinguished from the biological contrast and biological-sample identity. In GSE249479, condition and library-related labels were confounded; in GSE211713, mouse, radiation dose, and time were biological design variables, and no independent technical batch was documented. These variables were therefore not used as batch-removal covariates.

Cross-representation consensus and leave-one-representation-out sensitivity were computed using scgeo.tl.representation_stability(). Neighborhood overlap, edge retention, and normalized local-distortion diagnostics were computed using scgeo.tl.local_geometry_stability(). Controlled representation-corruption and local-distortion results are shown in Fig. S2, and the corresponding public-data sensitivity analyses are shown in Figs. S4–S8 and Tables S4–S6.

## 3 Results

### 3.1 Composition drift in the GSE280305 radiation-recovery representation

In the original GSE280305 case study, cluster-composition analysis revealed heterogeneous changes in cellular abundance between D8 and D21. Several clusters expanded or contracted during the recovery interval, as shown by the cluster-wise fractional changes and condition-specific compositions in Fig. 2B. Figure 2B summarizes cluster-composition drift between D8 and D21.

The Scanorama (Hie *et al.* 2019) representation retained recognizable annotated hematopoietic states and was used as the coordinate system for the original displacement and geometry–dynamics analyses. We therefore treat it as one case-study representation rather than as a uniquely valid or demonstrably optimal embedding. Robustness beyond this coordinate system was evaluated separately through cross-representation analyses in the independent validation workflows.

### 3.2 Controlled benchmarks define strengths and failure modes

The prespecified synthetic benchmark evaluated ScGeo under controlled perturbations with known truth and a held-out evaluation set. Robust estimators reduced sensitivity to outlier contamination relative to arithmetic mean displacement, although estimator performance differed under balanced biological-sample heterogeneity. Representation-corruption scenarios showed strong global detection of unstable representations, while state-level localization was more variable. In particular, pair-by-state localization of explicitly introduced local distortion remained limited, providing a retained negative result rather than evidence for reliable fine-grained localization. Controlled dynamics simulations correctly distinguished aligned from discordant geometry–dynamics relationships. Batch–condition confounding remained non-identifiable without additional design information.

These results establish the intended scope of ScGeo: it can improve robustness and make representation instability visible, but it cannot resolve confounded designs or guarantee precise localization of every geometric defect.

Under controlled outlier contamination, robust center estimators reduced displacement-magnitude error relative to arithmetic-mean displacement. The trimmed mean and geometric median showed the lowest held-out errors, whereas the coordinate-wise median also improved resistance to contamination. Performance differed under balanced biological-sample heterogeneity, however: the coordinate-wise median produced more false non-neutral classifications than the arithmetic mean or trimmed mean. These results indicate that robustness to outliers does not guarantee calibration under every sampling structure and support prespecifying the estimator together with biological sample aware uncertainty rather than adopting one universal center definition. Detailed benchmark results are presented in Figs. S1 and S2 and Table S2.

### 3.3 Post-irradiation redistribution of hematopoietic state space

**Figure 3:**
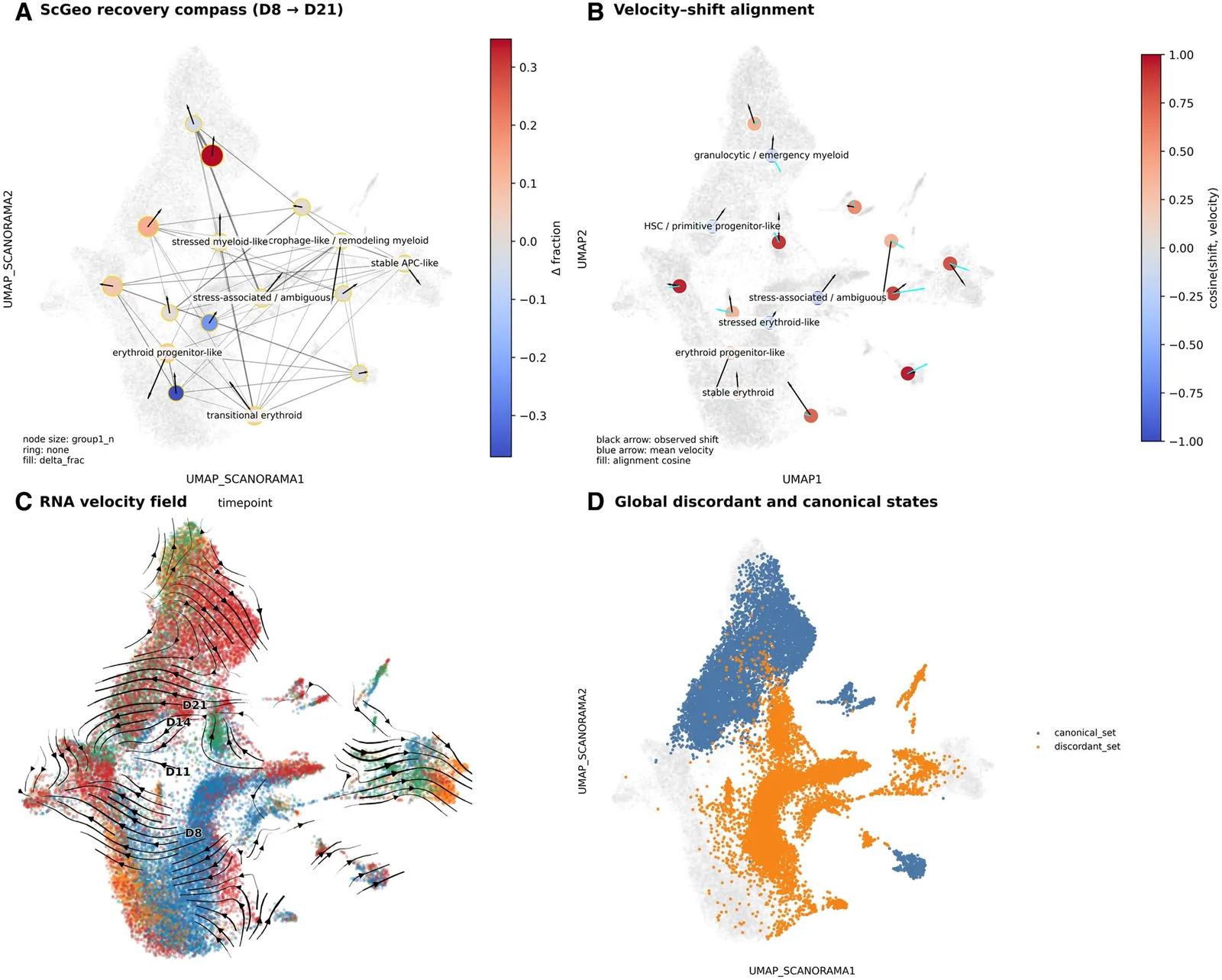
Geometry–velocity comparison reveals discordant recovery dynamics. (A) ScGeo recovery compass (D8 → D21). Cluster-level geometric displacement vectors (Δ-shift) projected onto the embedding. Node color indicates fractional abundance change; edges represent relationships between clusters. (B) Velocity–shift alignment. Comparison between geometric displacement vectors and mean RNA velocity vectors for each cluster. Color indicates cosine similarity, with negative values indicating discordance. Black arrows: observed geometric shift; blue arrows: mean RNA velocity. (C) RNA velocity field. Velocity streamlines overlaid on the embedding, showing inferred transcriptional dynamics across timepoints. (D) Alignment-based visualization of canonical and discordant categories. Cells inherit the alignment category of their annotated cluster according to the cluster-level comparison in panel B. The categories summarize agreement with the supplied velocity estimate and do not establish distinct causal trajectories.

We next quantified D8-to-D21 remodeling using cluster-specific geometric displacement vectors. For each annotated cluster g, the displacement vector was defined as the difference between its D21 and D8 centers in the analyzed representation, $\Delta_{g}={\hat{\mu }}_{g,D21}-{\hat{\mu }}_{g,D8}$. Figure 3A displays these vectors from the corresponding D8 cluster centers; arrow orientation represents displacement direction, and arrow length represents magnitude. The clusters showed heterogeneous displacement magnitudes and directions, with several populations exhibiting pronounced shifts. No single global directional-coherence statistic was calculated, and the analysis does not imply movement of all cells along a shared trajectory.

Using D8 as the post-irradiation reference condition, the fraction of cells exceeding the fixed reference-deviation threshold increased across the later sampling times (Fig. 2D). This pattern indicates progressive departure from the D8 reference distribution over the sampled interval. Because no untreated condition was included in this reference analysis, it does not establish departure from or return toward a healthy baseline.

Together, the composition, displacement, and D8-reference analyses show structured differences across the sampled post-irradiation interval without implying return toward an untreated baseline.

### 3.4 Embedding geometry captures recovery directionality beyond RNA velocity

To determine whether geometric displacement corresponds to transcriptional dynamics, we compared cluster-level displacement vectors (Δg) with RNA velocity vectors. While some clusters showed positive alignment between velocity and displacement, a substantial fraction exhibited low or negative cosine similarity, indicating disagreement between geometric shift and velocity directionality (Fig. 3B).

RNA velocity fields captured local transcriptional flow (Fig. 3C), whereas cluster-level D8-to-D21 displacement summarized observed differences between sampled conditions. Based on the sign and magnitude of cluster-level cosine alignment, cells inherited an alignment category from their annotated cluster for visualization in Fig. 3D. These categories describe agreement with the supplied velocity estimate and do not establish that discordant cells follow a separate causal trajectory.

The incomplete agreement between cluster displacement and RNA velocity indicates that the two summaries capture non-identical aspects of the data. Because RNA velocity is model-dependent and the geometric comparison is representation-dependent, discordance is interpreted as a hypothesis-generating signal rather than evidence that either method is incorrect.

### 3.5 Discordant states define biologically distinct programs of stress adaptation and lineage remodeling

**Figure 4:**
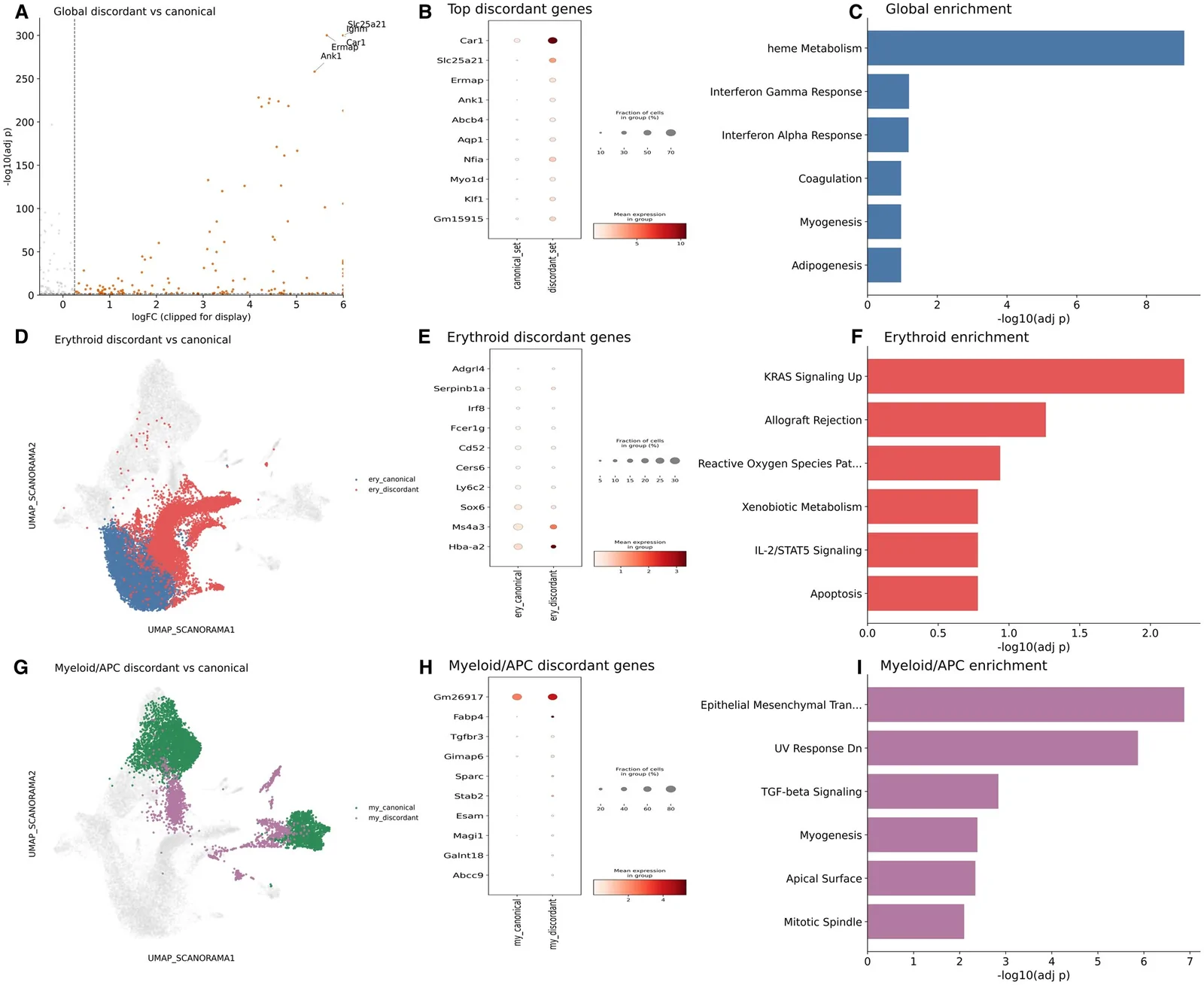
Geometry–velocity discordance is associated with stress and lineage-specific transcriptional programs. (A) Global differential expression (discordant vs canonical). Volcano plot showing differentially expressed genes between discordant and canonical states. (B) Top discordant-associated genes. Dot plot showing expression and prevalence of representative genes enriched in discordant states. (C) Global pathway enrichment. Enriched biological processes in discordant states, including heme metabolism, interferon response, and stress-related pathways. (D) Erythroid lineage: discordant vs canonical states. Spatial distribution of erythroid cells classified by alignment status. (E) Erythroid discordant genes. Dot plot of genes enriched in erythroid discordant states. (F) Erythroid pathway enrichment. Enrichment of pathways including ROS response, IL2/STAT5 signaling, and stress-related programs. (G) Myeloid/APC lineage: discordant vs canonical states. Spatial distribution of myeloid cells classified by alignment status. (H) Myeloid/APC discordant genes. Dot plot of genes enriched in discordant myeloid states. (I) Myeloid/APC pathway enrichment. Enrichment of pathways including TGFβ signaling, epithelial–mesenchymal transition (EMT)-like programs, and UV response.

To characterize transcriptional programs associated with geometry–velocity discordance, we compared expression and pathway enrichment between alignment categories. Discordant-category cells were enriched for heme metabolism, interferon response, and stress-related processes (Fig. 4A–C). Erythroid discordant-category cells showed ROS-response and IL2/STAT5-associated programs (Fig 4D–F), whereas myeloid/APC discordant-category cells showed TGFβ, EMT-like, and UV-response signatures (Fig. 4G–I). These associations support biological interpretation of the categories but do not exclude technical or model-derived contributions.

### 3.6 Geometry–velocity comparison highlights non-canonical recovery patterns

Taken together, the GSE280305 analysis identifies cluster-specific post-irradiation displacement that is only partially aligned with the supplied RNA-velocity field. The associated transcriptional programs motivate the term non-canonical in this study, while the analysis does not infer a new pseudotime, lineage tree, or causal recovery path.

### 3.7 ScGeo distinguishes representation-stable from representation-sensitive perturbation geometry

We next tested whether perturbation-associated conclusions in the validation workflows were reproducible across latent representations rather than specific to one coordinate system. PCA30, PCA50, and scVI formed the primary ensemble; PCA20 and diffusion maps were sensitivity views. UMAP was excluded from quantitative displacement and distributional calculations in these validation workflows.

In GSE249479, primary-representation state-effect rankings were highly concordant for TNF (Spearman ρ = 0.964) and LPS (ρ = 0.929). Activated HSC and Lymphoid states met the prespecified stable-effect criterion for TNF, whereas all seven evaluated states met it for LPS. The diffusion representation showed a median distortion of 0.591 relative to PCA50 and was retained as exploratory sensitivity; excluding it changed normalized effects by at most 0.016. Because biological-replicate identity was unavailable, all findings remained descriptive.

In GSE211713, representation consensus materially changed interpretation. All six major compartments were representation-unstable for control versus early 17 Gy. For control versus late 17 Gy, five compartments were stable and Fibroblast/stromal remained unstable; all six were stable for the early-versus-late 17 Gy cross-sectional contrast. The three primary comparisons used 5-versus-4, 5-versus-4, and 4-versus-4 independent mice, respectively, with 500 biological-mouse bootstrap resamples and exact assignment permutations.

These results illustrate the purpose of the representation-aware design: agreement is reported when conclusions persist across prespecified quantitative representations, while embedding-dependent findings remain explicitly labeled as representation-sensitive rather than being interpreted as robust biological effects. Supporting representation-quality, sensitivity, and mouse-level uncertainty analyses are presented in Figs. S4–S8 and Tables S4–S6.

### 3.8 Public validation extends ScGeo across biological systems

In the GSE132188-derived pancreatic-development workflow, prespecified developmental transitions generally showed concordant geometry–dynamics relationships across analyzed representations, while selected transitions showed weaker diffusion-based support. This workflow was treated as descriptive because the compared dynamics estimates were not independent experimental ground truth. The transition-level results and controls are presented in Fig. S3 and Table S3.

In GSE249479, 34 432 cells passed the prespecified quality-control workflow. TNF-associated stable descriptive effects were concentrated in Activated HSC and Lymphoid states, while LPS produced stable descriptive effects across all seven evaluated states. The HSC/quiescent LPS response was abundance-dominant: its abundance increased by 0.187, whereas normalized displacement was 0.284 and ranked sixth of seven states. Official R Augur and ScGeo rankings were partially concordant (TNF ρ = 0.750; LPS ρ = 0.393), consistent with their different estimands and the limited seven-state annotation. The principal analysis, representation-sensitivity checks, controls, and Augur comparison are presented in Figs. S4 and S5 and Table S4.

In GSE211713, 131 157 cells from 20 independent mouse libraries passed the prespecified workflow. Early control-versus-17 Gy effects were representation-sensitive in all six major compartments. Late effects were representation-stable in five compartments, with Fibroblast/stromal remaining unstable, and all six compartments showed stable early-versus-late cross-sectional differences. Fibroblast-subtype results were more sensitive: early-versus-late effects were stable for Col13a1-like and myofibroblast states but unstable for the Col14a1-like state. The replicate-aware major-state results, mouse-level uncertainty, representation sensitivity, and fibroblast-subtype analyses are presented in Figs. S6–S8 and Tables S5–S6.

Together, these workflows demonstrate that ScGeo can be applied across developmental, inflammatory, and radiation-response settings while preserving dataset-specific inference limits and negative findings.

## 4 Discussion and conclusion

Low-dimensional representations are foundational to single-cell analysis, but perturbation-associated geometry is often interpreted without explicit evaluation of estimator choice, biological sampling, local distortion, or representation dependence. ScGeo addresses this gap as a representation-aware downstream framework rather than as a new latent model or a claim of novelty for each constituent statistic.

Applied to the GSE280305 post-irradiation time series, ScGeo revealed heterogeneous cluster displacement and incomplete geometry–velocity agreement.

RNA velocity and ScGeo answer different questions: velocity estimates transcriptional directionality from kinetic assumptions, whereas ScGeo summarizes observed condition-dependent geometry and its robustness. Their comparison is therefore complementary but remains conditional on both the dynamics model and the supplied representation.

Recent methods use latent-variable models, foundation-model embeddings, and task-specific learning to construct biologically informative representations, including iVAE (Fu *et al*. 2025a), reinforcement-learning approaches to fate evaluation (Fu *et al*. 2025b), and scPASI. These approaches learn representations or phenotype-associated structures, whereas ScGeo evaluates perturbation-associated geometry and its stability within one or more supplied representations.

The expanded validation strategy addresses both controlled method behavior and biological portability. Synthetic benchmarks establish where the framework succeeds and where it remains limited; the pancreatic-development workflow evaluates geometry–dynamics relationships in a distinct developmental setting; GSE249479 tests descriptive perturbation geometry in human HSPCs; and GSE211713 evaluates replicate-aware radiation responses in lung tissue. These analyses indicate that ScGeo is not tied to one Scanorama embedding or one hematopoietic dataset.

At the same time, broader applicability should not be interpreted as universal validity. The conclusions depend on experimental design, biological replication, state annotation, representation quality, and coverage. GSE249479 remains descriptive because no biological-replicate identity was recoverable, and GSE211713 supports cross-sectional association rather than longitudinal or causal inference. The representation-sensitive early radiation findings and limited synthetic localization performance further show that the framework can expose uncertainty rather than eliminate it.

Integration methods and representation-learning methods construct latent coordinate systems, whereas ScGeo evaluates perturbation-associated conclusions drawn from those coordinate systems. ScGeo does not require batch correction and should not be interpreted as a competitor to Harmony (Korsunsky *et al*. 2019, Antonsson and Melsted 2025), Scanorama, scVI, or related methods. Instead, these methods may provide candidate representations whose local geometry and biological conclusions can be compared.

These analyses further show why integration quality cannot be reduced to condition mixing. When biological condition is confounded with library or is itself the signal of interest, maximizing condition mixing may remove relevant structure rather than preserve it. We therefore applied no correction in validation datasets lacking a genuine non-condition technical batch and assessed robustness across uncorrected or independently learned quantitative representations instead.

This approach does not identify a universally optimal embedding. Instead, it makes representation dependence observable by distinguishing conclusions that persist across prespecified spaces from those that change with dimensionality, latent model, or neighborhood geometry.

In GSE280305, geometry–velocity discordance was associated with stress-response and immune-activation programs. These associations motivate biological hypotheses about stressed hematopoiesis but do not prove that the categories are free of technical or modeling effects.

Robust center estimators reduced sensitivity to outlier contamination, but their behavior differed under replicate heterogeneity; ScGeo therefore records estimator choice and emphasizes biological-sample uncertainty rather than treating any single centroid definition as universally optimal.

ScGeo should be interpreted as a framework-level methodological contribution rather than as a claim of novelty for every constituent statistic. Euclidean displacement, distributional distances, neighborhood-overlap measures, and cosine alignment are established analytical concepts. ScGeo’s contribution is to combine these concepts with robust state estimators, biological-sample-aware uncertainty, cross-representation consensus, local-geometry diagnostics, and explicit evidence-status reporting. This organization makes representation dependence visible rather than assuming that a conclusion derived from one latent space is intrinsically biological.

ScGeo also differs in objective from methods that learn or predict cellular states. Optimal-transport and perturbation-prediction approaches estimate mappings or counterfactual distributions; trajectory-inference methods estimate progressions or branches; RNA velocity and CellRank estimate dynamical directionality; and integration methods construct corrected representations. ScGeo operates downstream of such methods and evaluates the robustness, geometric structure, and dynamical consistency of observed perturbation-associated differences.

The controlled benchmark and three public validation workflows suggest that ScGeo can be applied across developmental, inflammatory, hematopoietic, and radiation-response settings. Portability should nevertheless be judged within each study’s replication, annotation, representation quality, and inference scope rather than assumed a priori.

ScGeo is implemented as a modular, AnnData-compatible toolkit that records estimators, representations, coverage, uncertainty, and evidence status for reproducible downstream analysis (Virshup *et al*. 2024).

The enrichment of heme-metabolism and stress-response pathways among discordant-category cells is compatible with adaptive responses during stressed hematopoiesis. This interpretation remains hypothesis-generating; the present public dataset does not link these transient transcriptional states to long-term hematologic outcomes (Yoshida *et al*. 2021).

## Supplementary Material

vbag266_Supplementary_Data

## Data Availability

The public datasets analyzed in this study are available through the Gene Expression Omnibus under accessions GSE280305, GSE132188, GSE249479, and GSE211713. The pancreatic-development validation used a processed workflow derived from GSE132188. Synthetic benchmark definitions, configuration files, and held-out evaluation outputs are provided in the companion repository. Reproducible analysis workflows, including preprocessing, integration, geometric analysis, and figure-generation notebooks, are available at: https://github.com/liuifrec/scgeo-notebooks.

## Code availability

The ScGeo framework is available at: https://github.com/liuifrec/scgeo. Analysis workflows and reproducible notebooks used in this study are available at: https://github.com/liuifrec/scgeo-notebooks.

## Implementation

ScGeo is implemented in Python and is compatible with the Scanpy/AnnData ecosystem. The framework provides modular functions for computing geometric displacement, local mixing scores, distributional divergence, and velocity–displacement alignment. Results are stored within the AnnData object structure to facilitate integration with existing single-cell analysis workflows.
