# Supplementary material for: ScGeo reveals non-canonical trajectories beyond RNA velocity in radiation-induced hematopoietic recovery
Source: Bioinform Adv. 2026 Sep 9;6(1):vbag266. doi: 10.1093/bioadv/vbag266 (PMC13615651; doi:10.1093/bioadv/vbag266)
Supplement: Supplementary file 1 [file vbag266_supplementary_data.zip › Supplementary_Material.pdf]

ScGeo Supplementary Material

Supplementary Methods S1–S13 · Figures S1–S8 · Tables S1–S7

ScGeo reveals non-canonical trajectories beyond RNA velocity in radiation-induced hematopoietic recovery

Yu-Chen Liu · Kengo Yoshida

Radiation Effects Research Foundation, Hiroshima, Japan

Controlled simulations and public-data analyses evaluate estimator robustness, representation dependence, geometry-dynamics agreement, and biological-sample uncertainty across developmental, inflammatory, and radiation-response settings.

Contents

Contents....2

Supplementary Methods....4

Supplementary Method S1. Overview of validation analyses....4

Supplementary Method S2. Synthetic benchmark design and held-out evaluation....4

Supplementary Method S3. Robust state displacement and biological-sample uncertainty....4

Supplementary Method S4. Representation consensus, sensitivity roles, and leave-one-representation analysis....5

Supplementary Method S5. Local geometry, distributional diagnostics, and dynamics alignment....5

Supplementary Method S6. GSE132188-derived pancreatic-development workflow....6

Supplementary Method S7. GSE249479 preprocessing, representations, and descriptive ScGeo analysis. .6

Supplementary Method S8. Official R Augur comparison and implementation sensitivity analysis....7

Supplementary Method S9. GSE211713 quality control, annotation, and quantitative representations....7

Supplementary Method S10. GSE211713 primary and secondary cross-sectional contrasts....7

Supplementary Method S11. Biological-mouse bootstrap and exhaustive label permutations....8

Supplementary Method S12. Evidence classification and interpretation....8

Supplementary Method S13. Software and computational settings....9

Supplementary Figures....10

Figure S1. Synthetic benchmark: estimator robustness and biological-sample heterogeneity....10

Figure S2. Synthetic benchmark: representation corruption, local distortion, and geometry-dynamics stress tests....11

Figure S3. GSE132188-derived pancreatic-development geometry-dynamics validation....19

Figure S4. GSE249479 principal inflammatory HSPC analysis....23

Figure S5. GSE249479 quality, representation sensitivity, controls, and official R Augur comparison....25

Figure S6. GSE211713 replicate-aware radiation-response analysis and sample-level context....31

Figure S7. GSE211713 mouse-level bootstrap and exact permutation inference....38

Figure S8. GSE211713 representation stability, neutral findings, unstable findings, and fibroblast-subtype sensitivity....40

Supplementary Figure Legends and Alt Text....45

Figure S1. Synthetic benchmark: estimator robustness and biological-sample heterogeneity....45

Figure S2. Synthetic benchmark: representation corruption, local distortion, and geometry-dynamics stress tests....45

Figure S3. GSE132188-derived pancreatic-development geometry-dynamics validation..........................46

Figure S4. GSE249479 principal inflammatory HSPC analysis..47

Figure S5. GSE249479 quality, representation sensitivity, controls, and official R Augur comparison........47

Figure S6. GSE211713 replicate-aware radiation-response analysis and sample-level context..................48

Figure S7. GSE211713 mouse-level bootstrap and exact permutation inference.......................................49

Figure S8. GSE211713 representation stability, neutral findings, unstable findings, and fibroblast-subtype sensitivity..49

Supplementary Table Legends...50

Table S1. Datasets, designs, analysis units, and inference scope...50

Table S2. Synthetic benchmark estimates and retained failure modes..50

Table S3. GSE132188-derived pancreatic transition evidence..50

Table S4. GSE249479 descriptive state evidence and representation sensitivity.......................................51

Table S5. GSE211713 major-state primary evidence and mouse-level uncertainty...................................51

Table S6. GSE211713 neutral, unstable, and fibroblast-subtype evidence...51

Table S7. Estimands, parameters, software, and reproducibility..51

Data and code availability...51

Supplementary Methods

Supplementary Method S1. Overview of validation analyses

The supplementary analyses were designed to address three questions raised during review: whether ScGeo is robust to outliers and biological-sample variation, whether conclusions depend on the selected representation, and whether the framework can be applied beyond the original GSE280305 case study. We therefore combined controlled simulations with three public-data workflows. The synthetic benchmark evaluated estimator behavior, uncertainty, representation corruption, local distortion, and geometry-dynamics agreement under known conditions. The GSE132188-derived pancreatic-development workflow evaluated geometry-dynamics concordance in a developmental setting; GSE249479 evaluated inflammatory HSPC responses across multiple representations; and GSE211713 evaluated radiation-associated lung remodeling using independent mouse libraries.

Because the study designs support different levels of inference, each analysis was interpreted according to its available replication and comparison structure. The synthetic benchmark supports controlled method evaluation; the pancreatic-development and GSE249479 analyses are descriptive; and GSE211713 supports biological-sample-aware cross-sectional comparisons. The following sections describe the analysis settings and the results shown in Supplementary Figures S1-S8 and Tables S1-S7.

Supplementary Method S2. Synthetic benchmark design and held-out evaluation

The synthetic benchmark comprised 12 predefined scenarios evaluated with calibration seeds 0-4 and held-out seeds 5-19, yielding 240 independent jobs. Each job included four simulated biological samples per condition and 800 cells per biological sample. Calibration and held-out evaluation were summarized separately, and thresholds selected during calibration were not changed after inspection of the held-out results.

The evaluated center estimators were the arithmetic mean, a robust mean, the coordinate-wise median, a 10% trimmed mean, and the geometric median. The state-shift threshold was set to 0.5 before held-out evaluation. Bootstrap summaries used 300 iterations, and local-neighborhood diagnostics were evaluated at k = 15, 30, and 50. Representation-corruption, local-distortion, and aligned or discordant dynamics scenarios were used to test method behavior under known conditions.

The benchmark also identified important limitations. Under balanced biological-sample heterogeneity, the coordinate-wise median made at least one false non-neutral call in 8 of 15 held-out jobs, the geometric median in 2 of 15, and the other three estimators in 0 of 15. Mean held-out assessed-state bootstrap coverage was 0.805. In representation-corruption scenarios, held-out state-instability recall was 0.767, whereas fine-grained local-distortion recall was 0.

Supplementary Method S3. Robust state displacement and biological-sample uncertainty

For a state and condition, ScGeo summarizes the supplied quantitative coordinates with a selected center estimator. Condition-associated displacement is the vector between condition-specific state centers. A

normalized displacement divides the raw center distance by a within-state reference scale defined before evaluation, allowing dimensionless comparisons without combining displacement, abundance, and distributional distance into a single score.

Where independent biological samples were available, centers were computed for each biological sample before between-group comparison. Biological-sample bootstrap intervals resampled samples, not cells. GSE211713 used geometric-median mouse centers and 500 mouse-level bootstrap iterations. GSE249479 lacked a valid biological-replicate identifier; cell-level repeated subsampling there was labeled a computational stability control and did not produce biological uncertainty or inferential p-values.

Coverage requirements were evaluated before ScGeo estimation. GSE211713 major states required at least 30 cells per mouse and fibroblast subtypes at least 20 cells per mouse, with at least three eligible mice in each compared group. Ineligible or unavailable state/representation combinations were retained as such rather than imputed.

Supplementary Method S4. Representation consensus, sensitivity roles, and leave-one-representation analysis

Quantitative representations were assigned distinct analytical roles. For GSE249479 and GSE211713, PCA30, PCA50, and scVI formed the primary ensemble; PCA20 was used to assess dimensional sensitivity, and diffusion maps were treated as exploratory sensitivity. UMAP was used only for visualization. A stable-effect or stable-neutral conclusion required agreement across the primary ensemble under predefined consensus criteria; materially discordant results were labeled representation-sensitive.

Representation quality was evaluated using neighborhood overlap relative to PCA50, local-distance agreement, connectivity, and related diagnostics. Dose, time, or treatment mixing was not optimized because those variables encoded biological design. Leave-one-primary-representation calculations quantified the largest change caused by omitting one primary view. Exploratory diffusion results were reported as sensitivity analyses and did not override the primary-representation consensus.

For GSE211713, primary representation consensus materially changed interpretation: all six major compartments were unstable for control versus early 17 Gy, five of six were stable for control versus late 17 Gy with Fibroblast/stromal unstable, and all six were stable for the cross-sectional early-versus-late 17 Gy comparison. The dataset was therefore not described globally as robust to representation choice.

Supplementary Method S5. Local geometry, distributional diagnostics, and dynamics alignment

Local geometry diagnostics compared nearest-neighbor relationships at k = 15, 30, and 50, with exact calculations capped at 3,000 cells where required by the analysis settings. These diagnostics assessed whether local relationships were preserved across representations; they did not establish that any representation was biologically definitive. Synthetic tests showed that global corruption could be detected, whereas fine-grained state-local distortion remained difficult to localize.

Energy distance, maximum mean discrepancy, and sliced Wasserstein distance were reported as distributional descriptions with their comparison unit stated. GSE249479 used within-state balanced cell samples of at most 600 cells per state and condition, 64 sliced-Wasserstein projections, and a 250-cell MMD pilot. GSE211713 displayed pooled-cell and biological-mouse-center energy distances separately. None of these distances substituted for biological-sample inference.

When an externally supplied dynamics vector was available, alignment was quantified as the cosine between the condition-associated displacement direction and the supplied direction. Values of at least +0.3 were classified as aligned and values of at most -0.3 as discordant; intermediate values were neutral. Combinations lacking a usable dynamics vector remained unavailable and were not counted as successful classifications.

Supplementary Method S6. GSE132188-derived pancreatic-development workflow

The public pancreatic-development workflow derived from GSE132188 contained 2,531 cells with original cluster annotations. Quantitative views comprised PCA20, PCA30, PCA50, and a 10-component diffusion representation; UMAP was used only for developmental visualization. Prespecified annotation-supported endocrine transitions were evaluated from Ngn3-low endocrine progenitors through Ngn3-high and Fev-positive precursors toward terminal endocrine states.

scVelo dynamical modeling used 50 principal components, 30 neighbors, random seed 0, max_iter = 25, n_jobs = 1, min_r2 = 0.01, and min_likelihood = 0.001. CellRank GPCCA used a forward VelocityKernel, deterministic/correlation settings, seed 0, 20 Schur vectors, 12 macrostates, four terminal states, one initial state, and the GMRES solver without PETSc. A source state required at least 20 cells, at least two usable representations, and an aligned fraction of at least 0.6 for the aligned consensus class.

Controls used reversed edges, shuffled velocity, and rotated velocity, with control seed 1729. CellRank was a complementary velocity-derived comparator, not independent experimental ground truth. Consequently, this workflow is descriptive and does not establish causal lineage transitions.

Supplementary Method S7. GSE249479 preprocessing, representations, and descriptive ScGeo analysis

GSE249479 was reconstructed from public GEO supplementary matrices for PBS, TNF, and LPS libraries. The sparse workflow retained cells with nonzero counts, more than 1,000 detected genes, and mitochondrial fraction below 18%; full cells-by-genes matrices were never densified. The final analyzed dataset contained 34,432 cells. Conservative marker-based annotation retained ambiguous and low-confidence labels: 10,154 cells were ambiguous and 10,272 were low confidence.

Counts were normalized to 10,000 per cell and log1p transformed. Three thousand highly variable genes were selected. Quantitative representations were PCA20, PCA30, PCA50, a 20-dimensional scVI latent representation (two hidden layers, 128 hidden units, dropout 0.1, negative-binomial gene likelihood, batch size 256, maximum 100 epochs, early-stopping patience 10, train fraction 0.9), and a 20-component

diffusion map built from 30 neighbors. No batch covariate was supplied to scVI because condition/library labels were confounded with the biological contrast.

ScGeo used the geometric median, 10% trimming where applicable, seed 1729, and the PCA30/PCA50/scVI primary ensemble. Ten repeated balanced-cell subsamples and shuffled-cell-label controls quantified computational stability only. Because no independent biological-replicate identity could be recovered, the workflow automatically used cells as a descriptive computational unit; all GSE249479 findings are descriptive.

Supplementary Method S8. Official R Augur comparison and implementation sensitivity analysis

Official R Augur version 1.0.3 was the primary external comparator for GSE249479. The analysis used 50 subsamples, 20 cells per condition per subsample, three-fold classification, upper-50% LOESS-residual variable features, a 50% random-feature fraction, 100 trees, and mtry = 2. Five seed blocks began at 1729, 1830, 1931, 2032, and 2133; shuffled-label blocks began at 11729, 11830, 11931, 12032, and 12133.

The retained Python Augur-inspired calculation used sparse raw-count variance selection and scikit-learn and was treated only as implementation sensitivity. Official and Python AUC values were not averaged, pooled, or merged. Augur treatment separability and ScGeo displacement quantify different estimands and were compared by rank without treating one as validation of the other.

Supplementary Method S9. GSE211713 quality control, annotation, and quantitative representations

GSE211713 comprised 20 independent female C57BL/6J mouse lung libraries spanning control, 10 Gy, and 17 Gy conditions at months 1-5 after exposure. Each GSM/mouse library was treated as a biological sample. The analysis retained 131,157 cells. Because the publication did not report the exact QC thresholds used for its final atlas, the thresholds used here were defined independently and were not adjusted to reproduce the publication cell count.

Cells were filtered using a minimum of 500 total counts, a minimum of 250 detected genes, a maximum mitochondrial fraction of 0.25, a minimum gene/count ratio of 0.05, and five-MAD extreme-library diagnostics. Per-mouse Scrublet analysis used an expected doublet rate of 0.06, a fixed score threshold of 0.25, a very-high score threshold of 0.50, seed 1729, and 30 principal components. Genes detected in at least three retained cells were kept, and 3,500 highly variable genes were selected using condition-blind sparse raw-count dispersion.

Counts were normalized to 10,000 per cell and log1p transformed. PCA20, PCA30, PCA50, 20-dimensional scVI, and a 20-component diffusion map were generated with seed 1729; primary and sensitivity roles matched Supplementary Method S4. Mouse, dose, and time were biological design variables, and no independent technical batch covariate was used for correction.

Supplementary Method S10. GSE211713 primary and secondary cross-sectional contrasts

The three prespecified primary contrasts were control versus early 17 Gy (months 1–2; five versus four mice), control versus late 17 Gy (months 4–5; five versus four mice), and early versus late 17 Gy (four versus four mice). Individual mice differed across times, so every temporal comparison is cross-sectional. Related contrasts reuse some control mice and are not independent.

Secondary all-17 Gy and all-10 Gy comparisons pooled heterogeneous post-exposure times and were labeled replicate-aware secondary, time-heterogeneous sensitivity analyses. Mouse-level abundance was reported separately from geometric displacement. Marker-inferred major and fibroblast-subtype annotations retained confidence and coverage qualifications.

The design supports associations between radiation group/time stratum and state geometry. It does not support within-mouse change, longitudinal trajectories, causal radiation effects, or proof that a representation preserves all biological structure.

Supplementary Method S11. Biological-mouse bootstrap and exhaustive label permutations

For each eligible GSE211713 state and representation, 500 bootstrap draws independently resampled mice within each comparison group and recomputed the normalized displacement. Stored raw and normalized 95% intervals, direction resultant length, direction stability, and sign stability were reported without replacing the representation-consensus classification.

Exact permutation inference enumerated all group-label assignments while preserving group sizes. The observed assignment was included. Upper-tail fractions counted assignment statistics greater than or equal to the observed statistic, including ties, and no plus-one correction was applied because the full assignment space was enumerated. The three primary designs contained 126, 126, and 70 assignments, with minimum attainable grids of 1/126, 1/126, and 1/70.

Within-contrast and representation Benjamini–Hochberg values were retained as sensitivity only. Coarse exact grids and shared mice across related contrasts were stated explicitly; permutation evidence was not converted into a claim of longitudinal change or causality.

Supplementary Method S12. Evidence classification and interpretation

Results were summarized as stable, stable-neutral, representation-sensitive, insufficient-coverage, unavailable, or descriptive according to the available evidence. Sensitivity analyses and negative findings are reported alongside positive results so that representation dependence and coverage limitations remain visible.

A high displacement observed in only one representation was not classified as stable when the primary ensemble disagreed. Cell-level repeated subsampling was treated as a computational stability analysis rather than biological replication. Distributional distance was not interpreted as a p-value, and geometry-

dynamics agreement was not treated as independent validation when both quantities arose from related velocity-based models.

GSE249479 was interpreted descriptively because no independent biological-replicate identifier was recoverable. GSE211713 used independent mouse libraries as biological samples but supports cross-sectional rather than longitudinal inference. The pancreatic workflow is described as a public pancreatic-development workflow derived from GSE132188.

Supplementary Method S13. Software and computational settings

Analyses were performed in Python 3.12.3. Dataset-specific configuration files record the package versions, analysis parameters, and random seeds used for each workflow. The principal seed was 1729 for representation and quality-control workflows, seed 0 was used for pancreas scVelo and CellRank fitting, and seed 1729 was used for pancreas control analyses. Bootstrap seeds were stored for each state and representation.

The ScGeo software and analysis notebooks are available through the repositories listed in the main manuscript. Supplementary Table S7 summarizes the principal estimands, thresholds, group sizes, software settings, and random seeds used in the analyses.

Numerical results are provided in Supplementary Tables S1-S7. Values are shown at practical precision in the manuscript-facing tables, while the accompanying workbook retains the underlying numeric values used for figure generation.

Supplementary Figures

Figure S1. Synthetic benchmark: estimator robustness and biological-sample heterogeneity

Supplementary Figure S1. Estimator robustness and simulated biological-sample heterogeneity

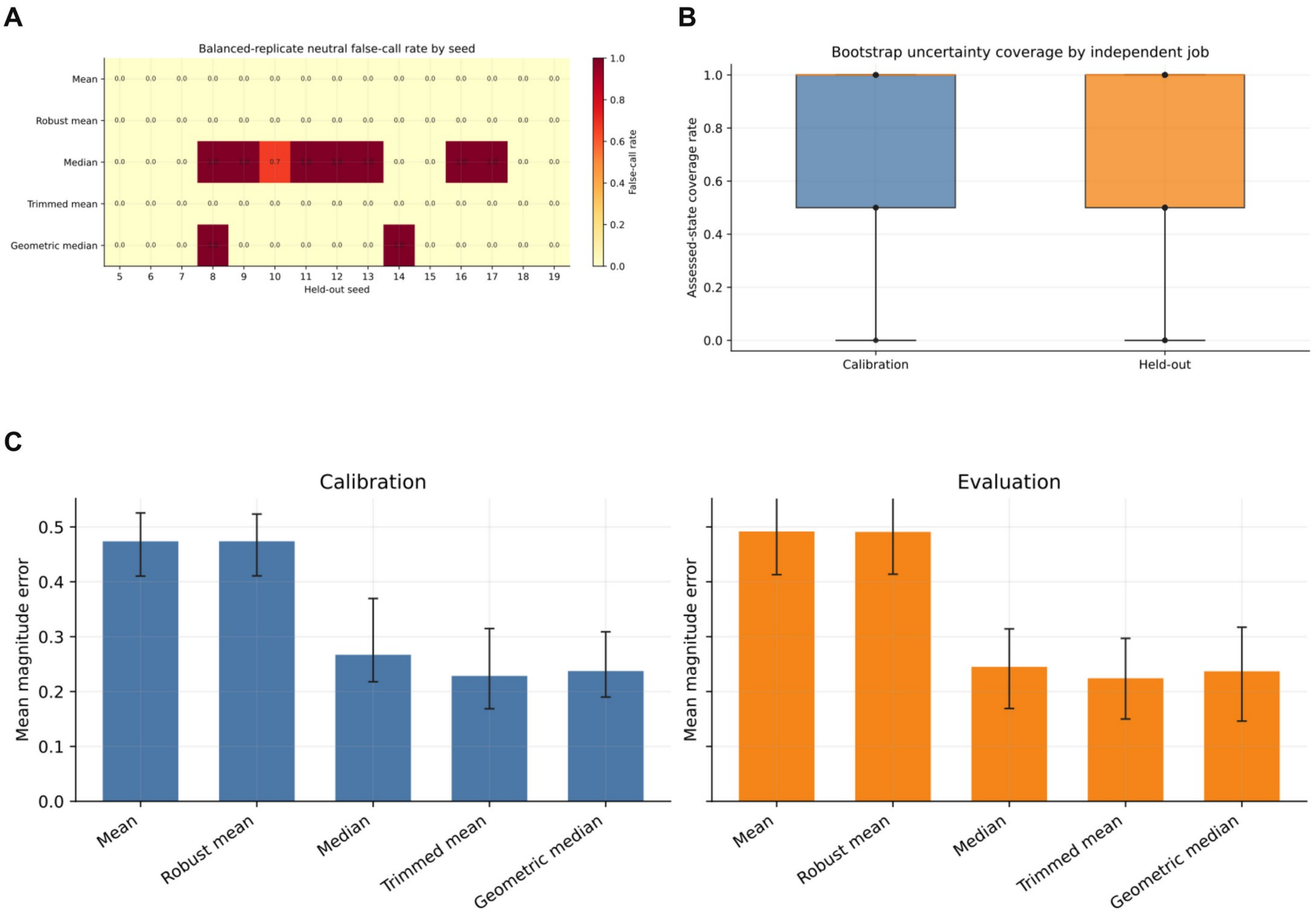

Figure S1

Figure S2. Synthetic benchmark: representation corruption, local distortion, and geometry-dynamics stress tests

Supplementary Figure S2. Representation corruption, local distortion, ablation results, and geometry-dynamics stress tests

A

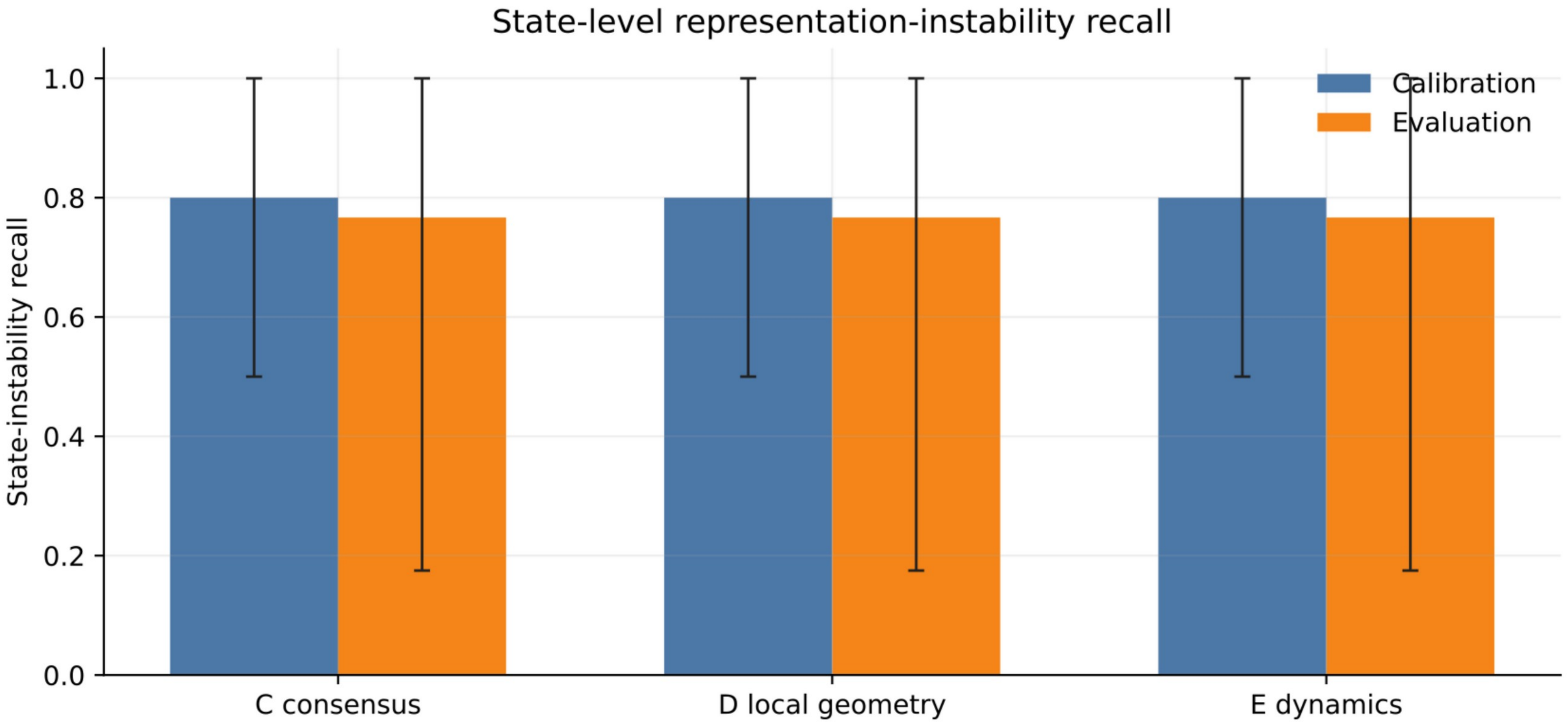

Figure S2 · band 1 of 8

Figure S2 (continued; band 2 of 8)

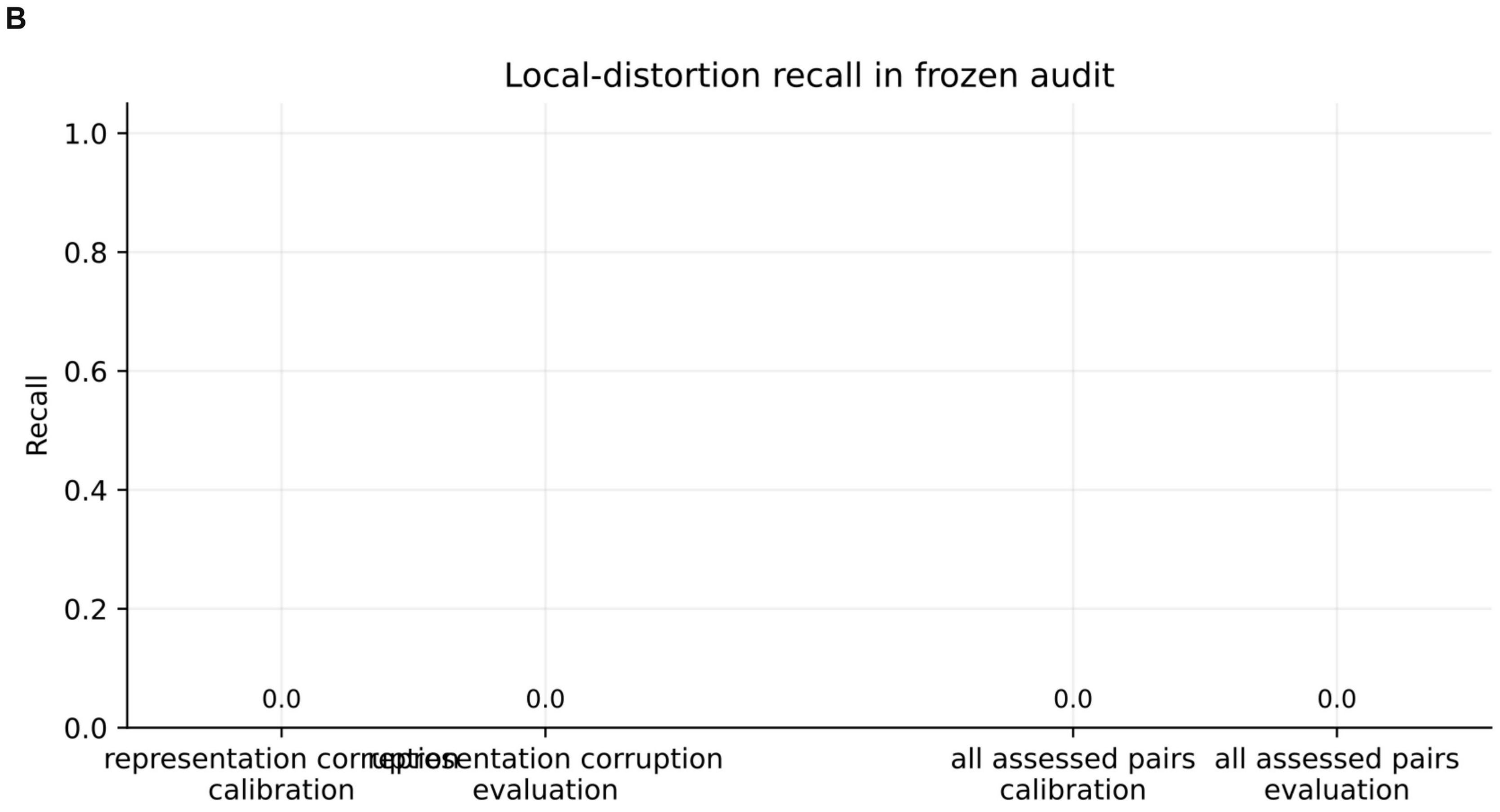

Figure S2 · band 2 of 8

Figure S2 (continued; band 3 of 8)

C

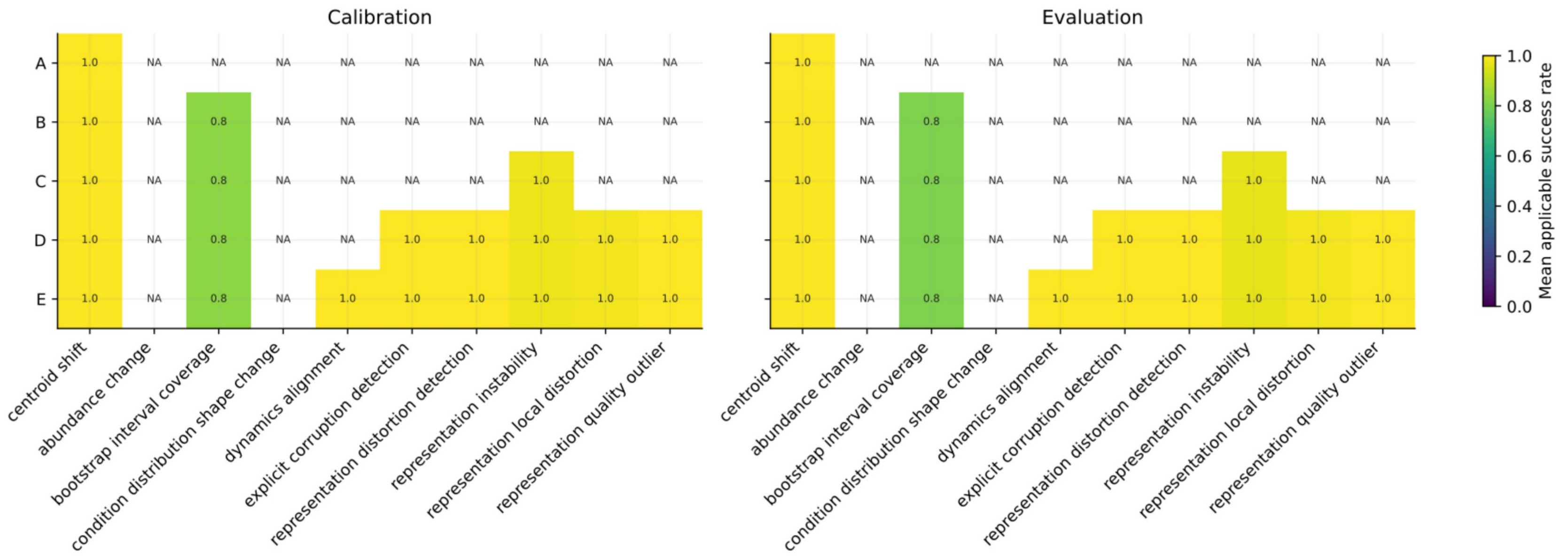

Figure S2 · band 3 of 8

Figure S2 (continued; band 4 of 8)

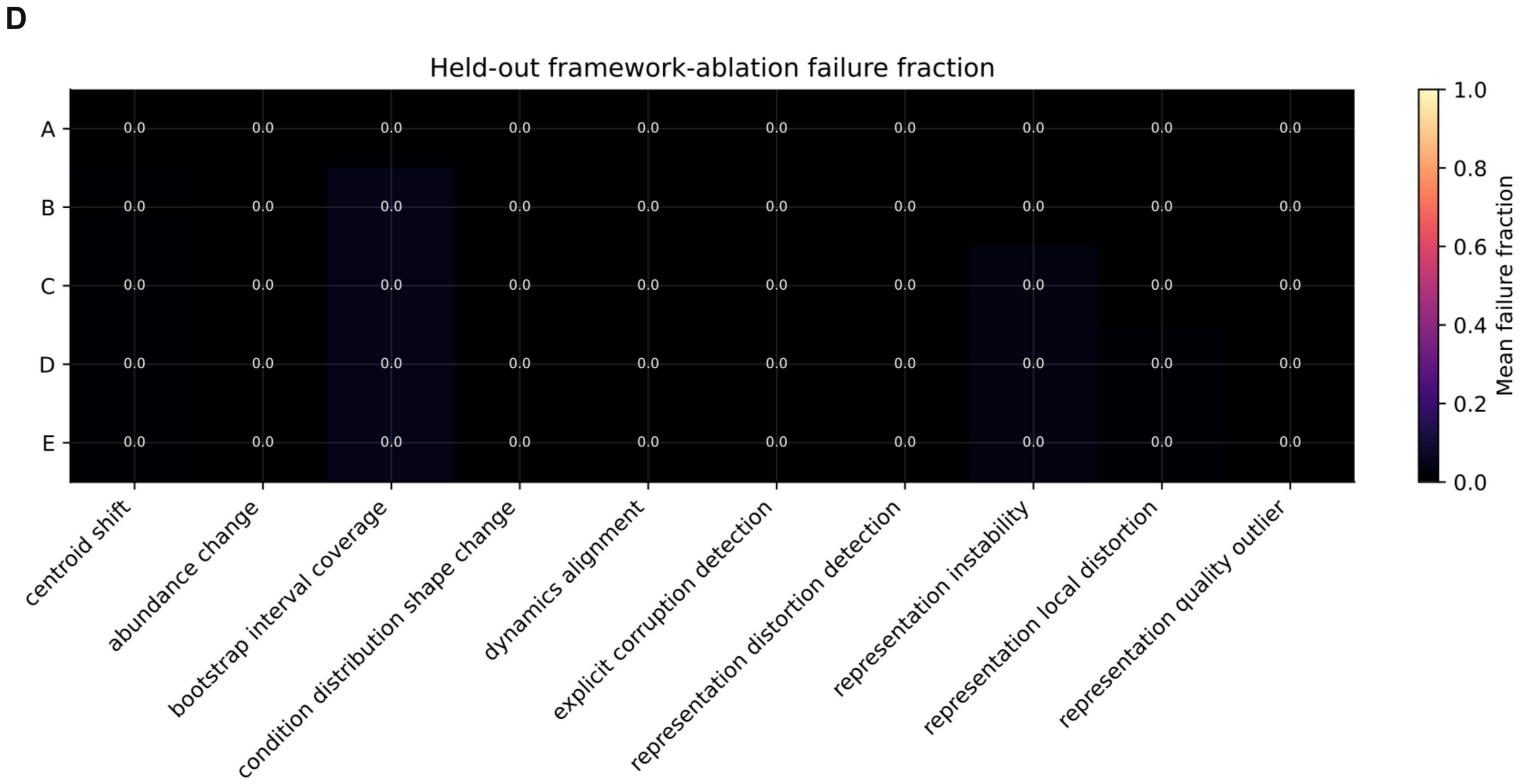

Figure S2 · band 4 of 8

Figure S2 (continued; band 5 of 8)

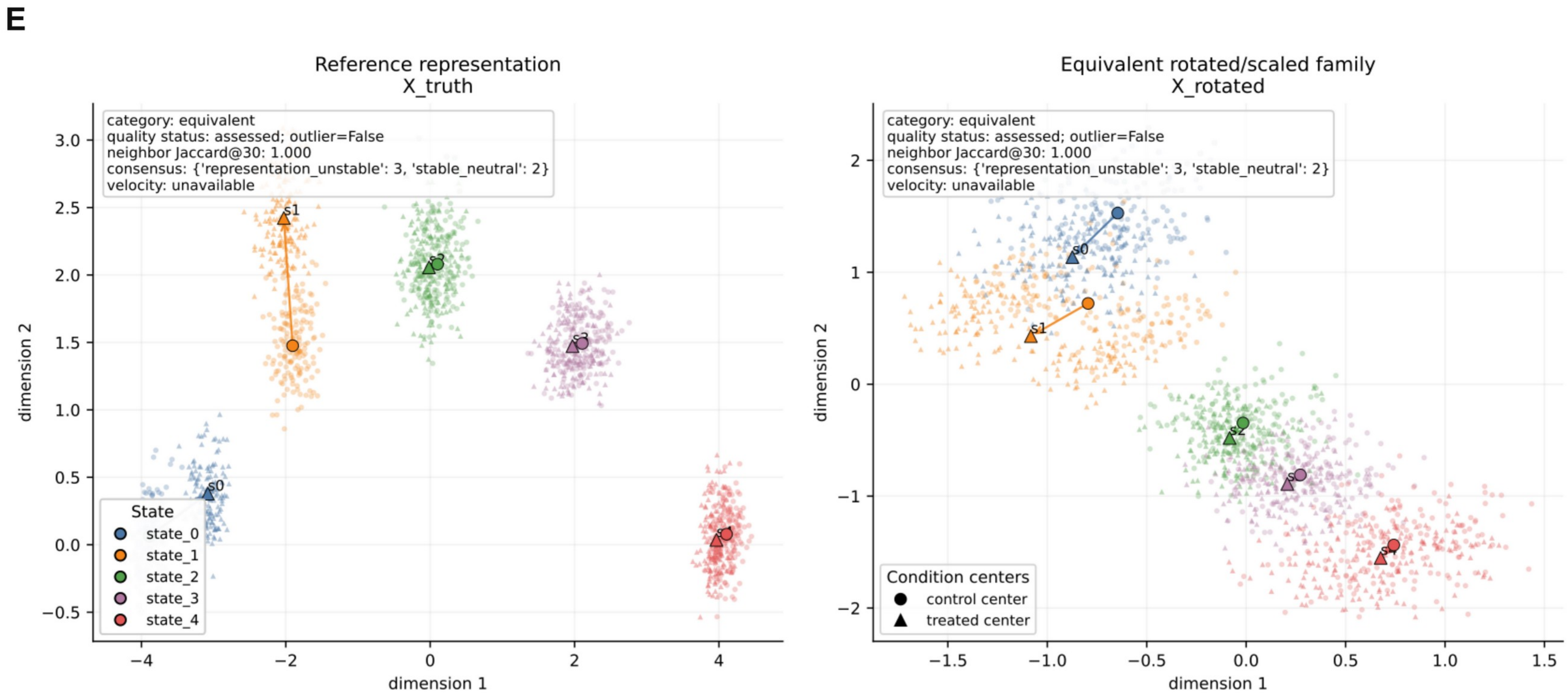

Figure S2 · band 5 of 8

Figure S2 (continued; band 6 of 8)

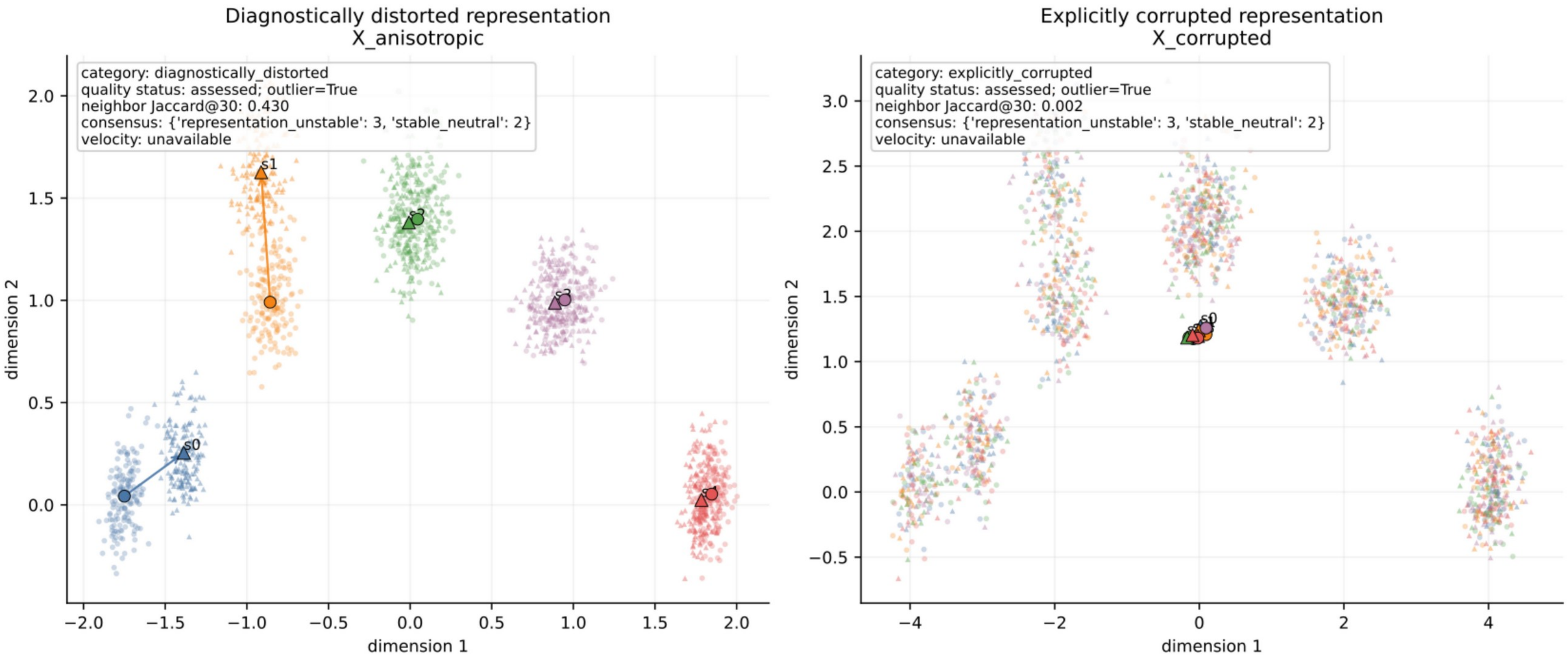

Figure S2 · band 6 of 8

Figure S2 (continued; band 7 of 8)

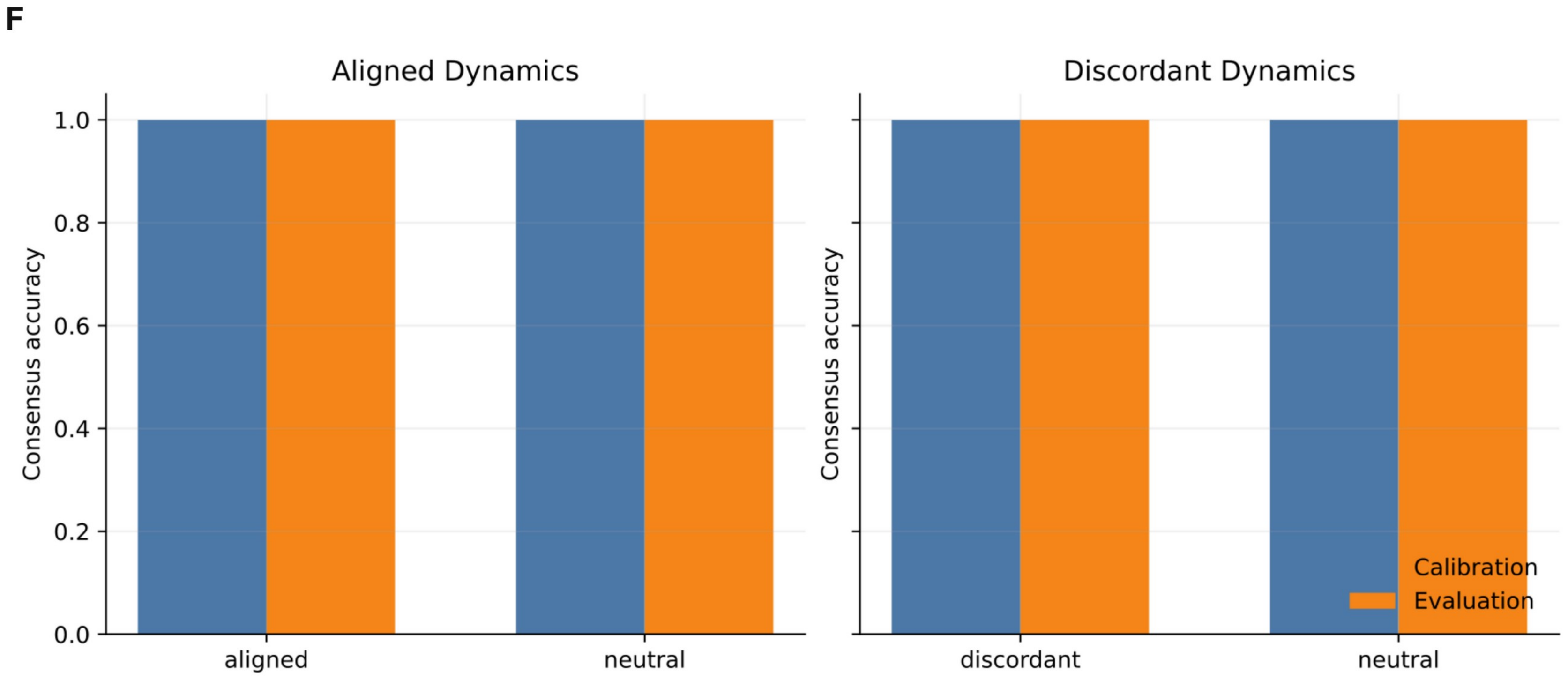

Figure S2 · band 7 of 8

Figure S2 (continued; band 8 of 8)

G

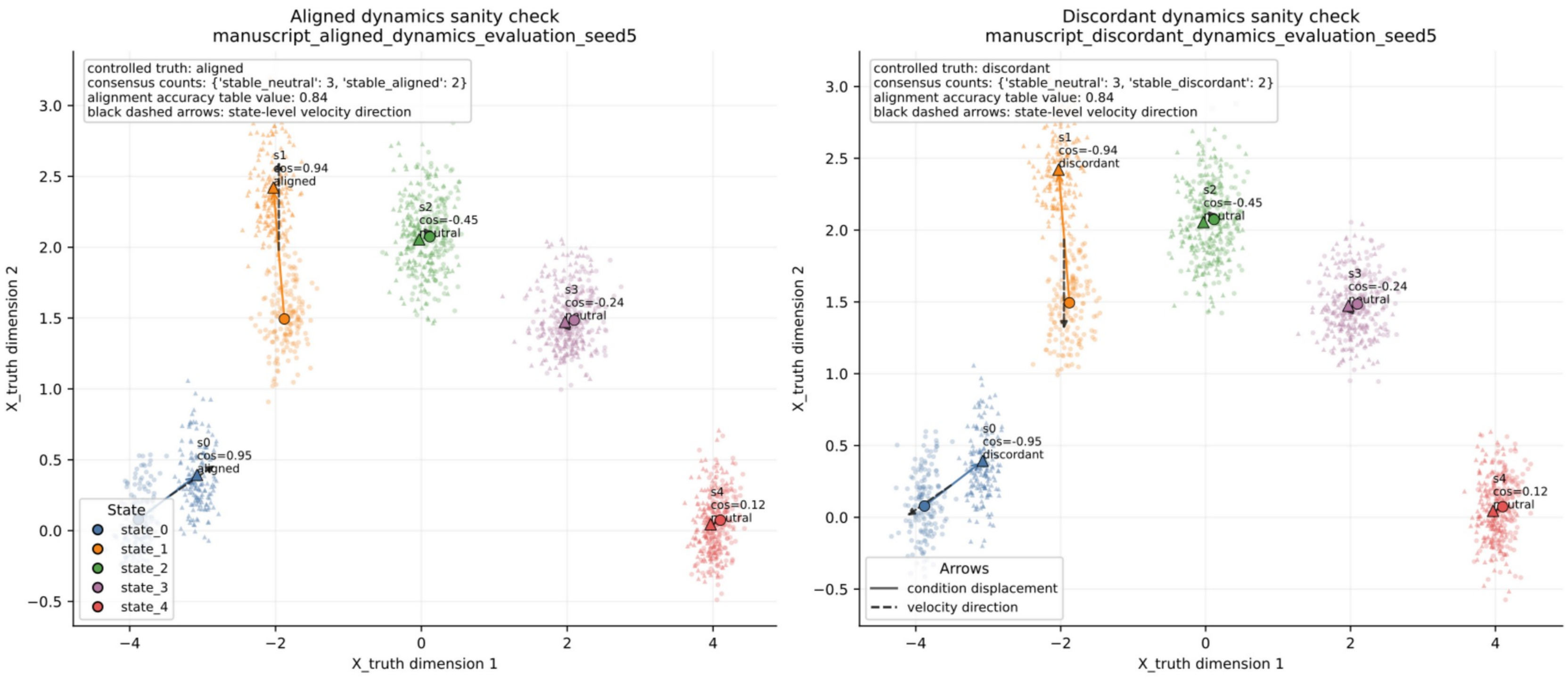

Figure S2 · band 8 of 8

Figure S3. GSE132188-derived pancreatic-development geometry-dynamics validation

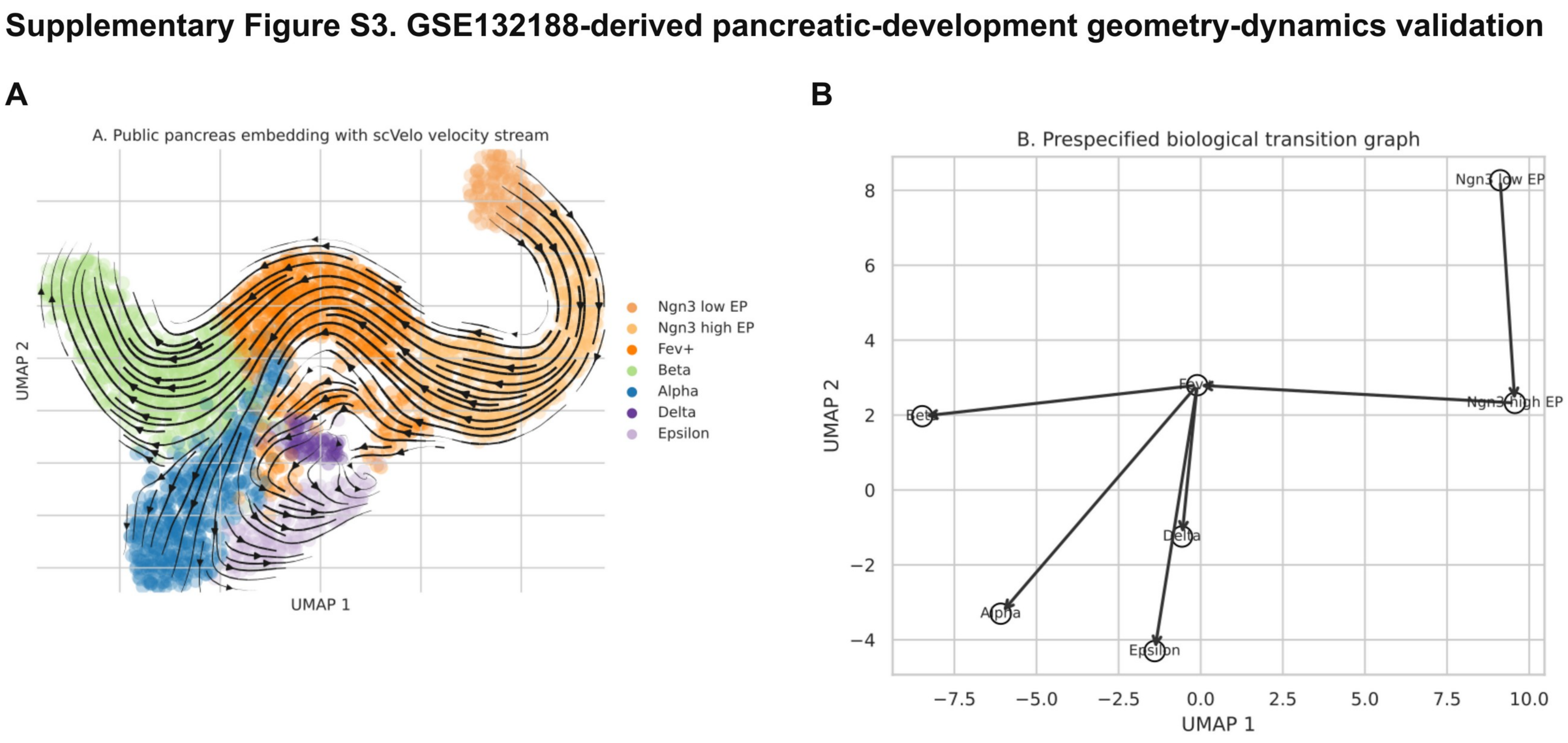

Figure S3 · band 1 of 4

Figure S3 (continued; band 2 of 4)

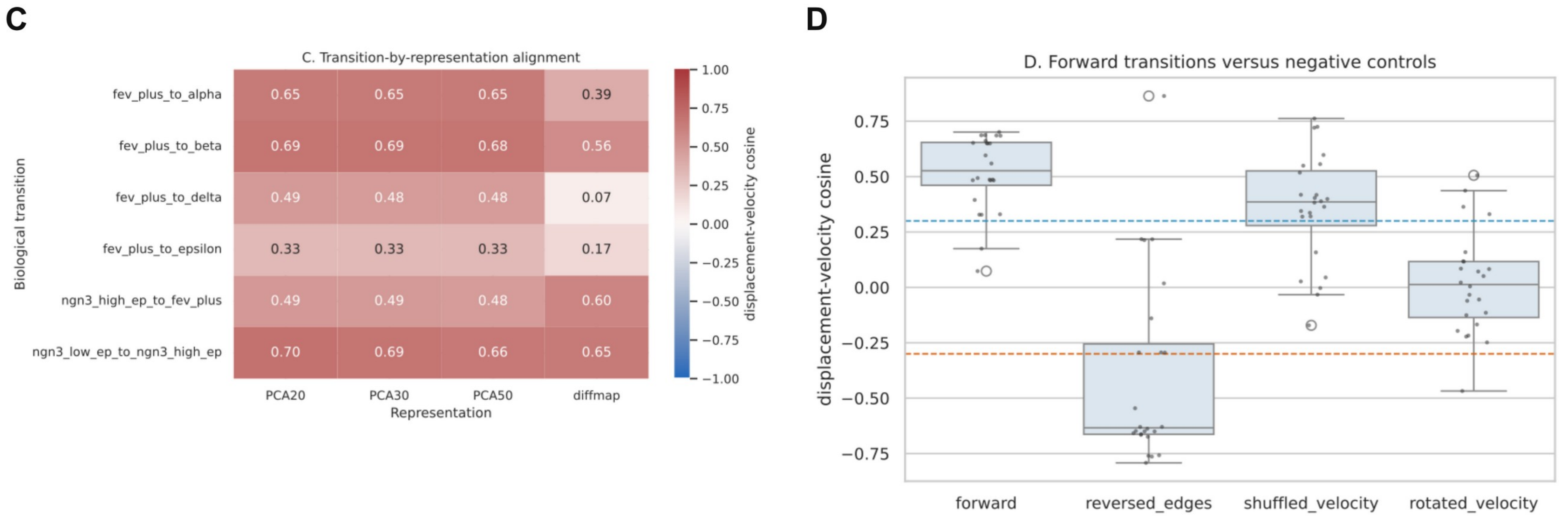

Figure S3 • band 2 of 4

Figure S3 (continued; band 3 of 4)

E

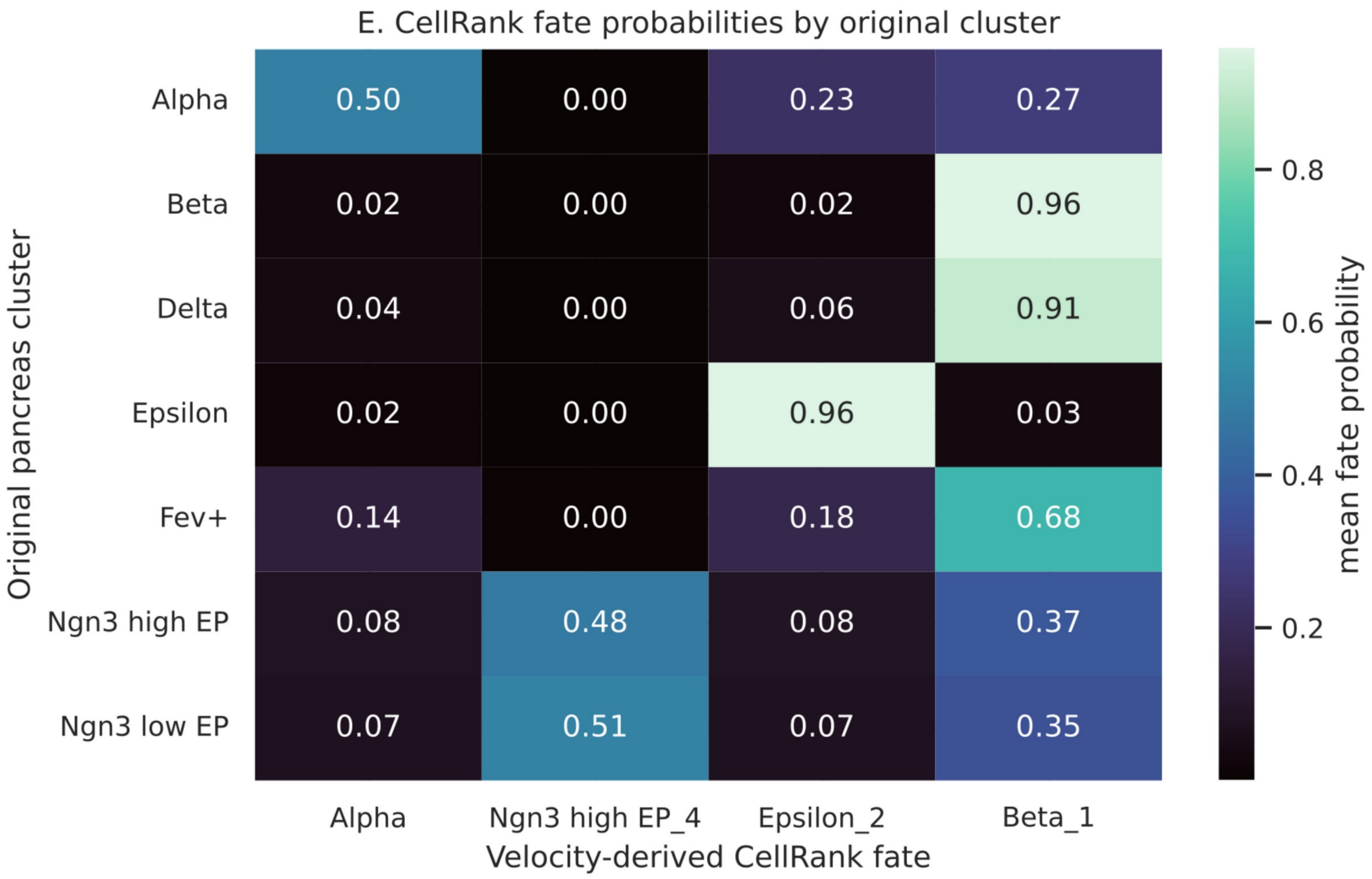

Figure S3 · band 3 of 4

Figure S3 (continued; band 4 of 4)

F

F. State-transition evidence table

transition	class	repr. agree	neg. specificity	CellRank	visible limitation
ngn3 low ep to ngn3 high ep	aligned	1.00	0.87	velocity-derived	negative controls retain aligned calls in 0.13 of tested representation-control...
ngn3 high ep to fev plus	aligned	1.00	0.53	unmatched fate	negative controls retain aligned calls in 0.47 of tested representation-control...
fev plus to alpha	aligned	1.00	0.67	velocity-derived	negative controls retain aligned calls in 0.33 of tested representation-control...
fev plus to beta	aligned	1.00	0.60	velocity-derived	negative controls retain aligned calls in 0.40 of tested representation-control...
fev plus to delta	aligned	0.75	0.67	unmatched fate	negative controls retain aligned calls in 0.33 of tested representation-control...
fev plus to epsilon	aligned	0.75	0.60	velocity-derived	negative controls retain aligned calls in 0.40 of tested representation-control...

Figure S3 · band 4 of 4

Figure S4. GSE249479 principal inflammatory HSPC analysis

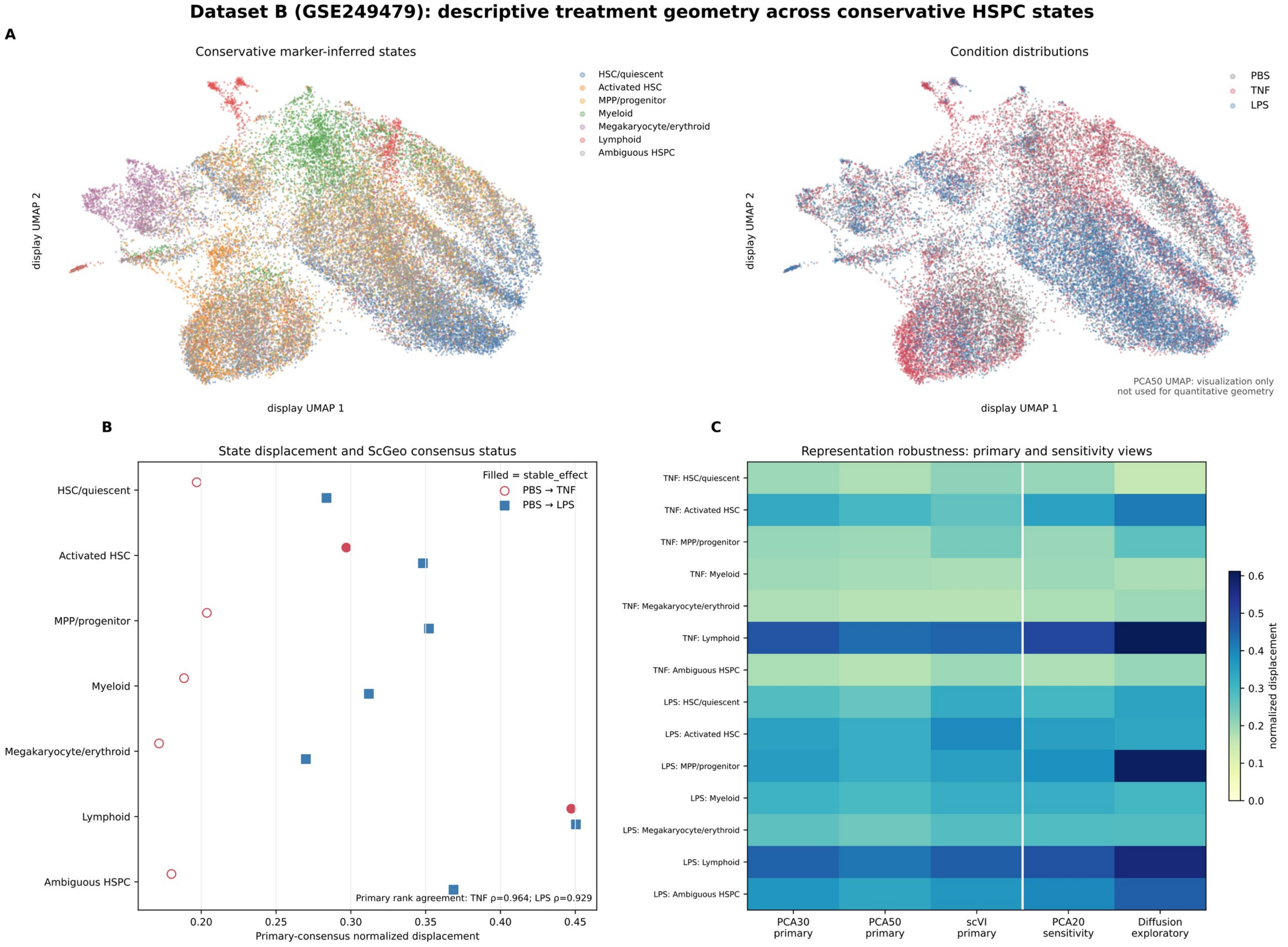

Figure S4 · band 1 of 2

Figure S4 (continued; band 2 of 2)

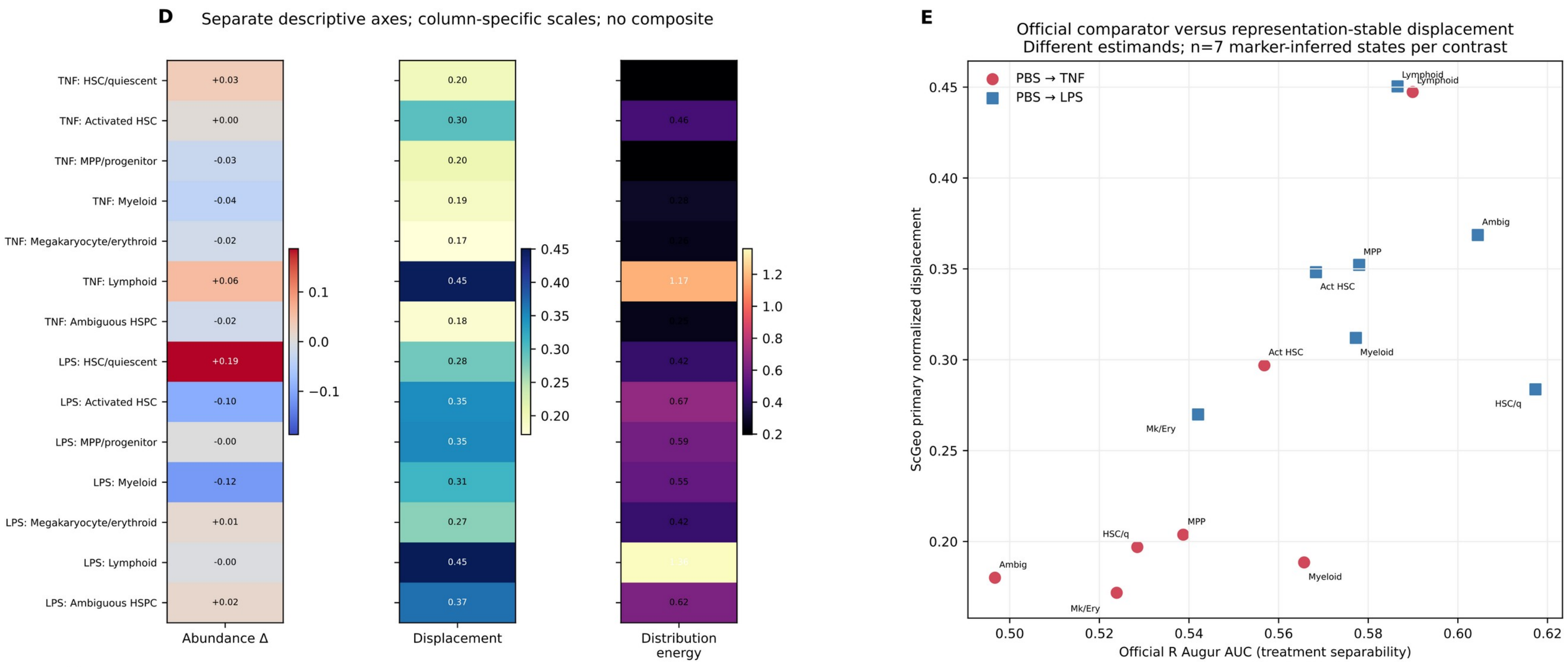

F

LIMITATION • descriptive_only • no independent biological replicate identity • cells, conditions, libraries, and SouporCell clades are not replicates

Figure S4 · band 2 of 2

Figure S5. GSE249479 quality, representation sensitivity, controls, and official R Augur comparison

Supplementary Figure S5. GSE249479 quality control, representation stability, controls, official R Augur comparison, and signature analyses

A

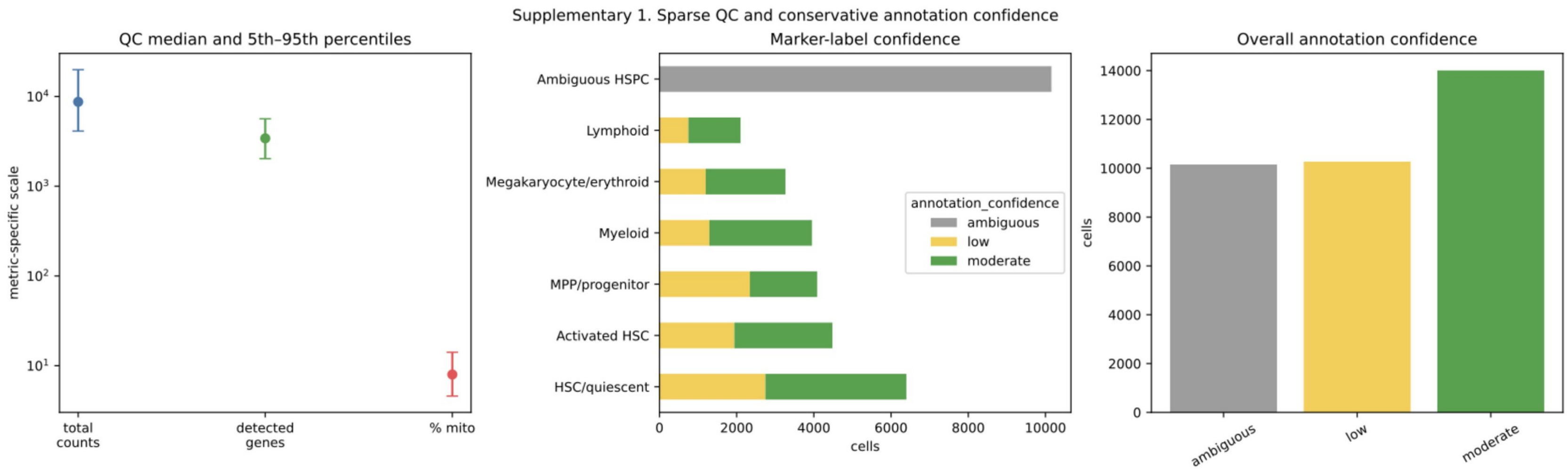

Figure S5 · band 1 of 6

Figure S5 (continued; band 2 of 6)

B

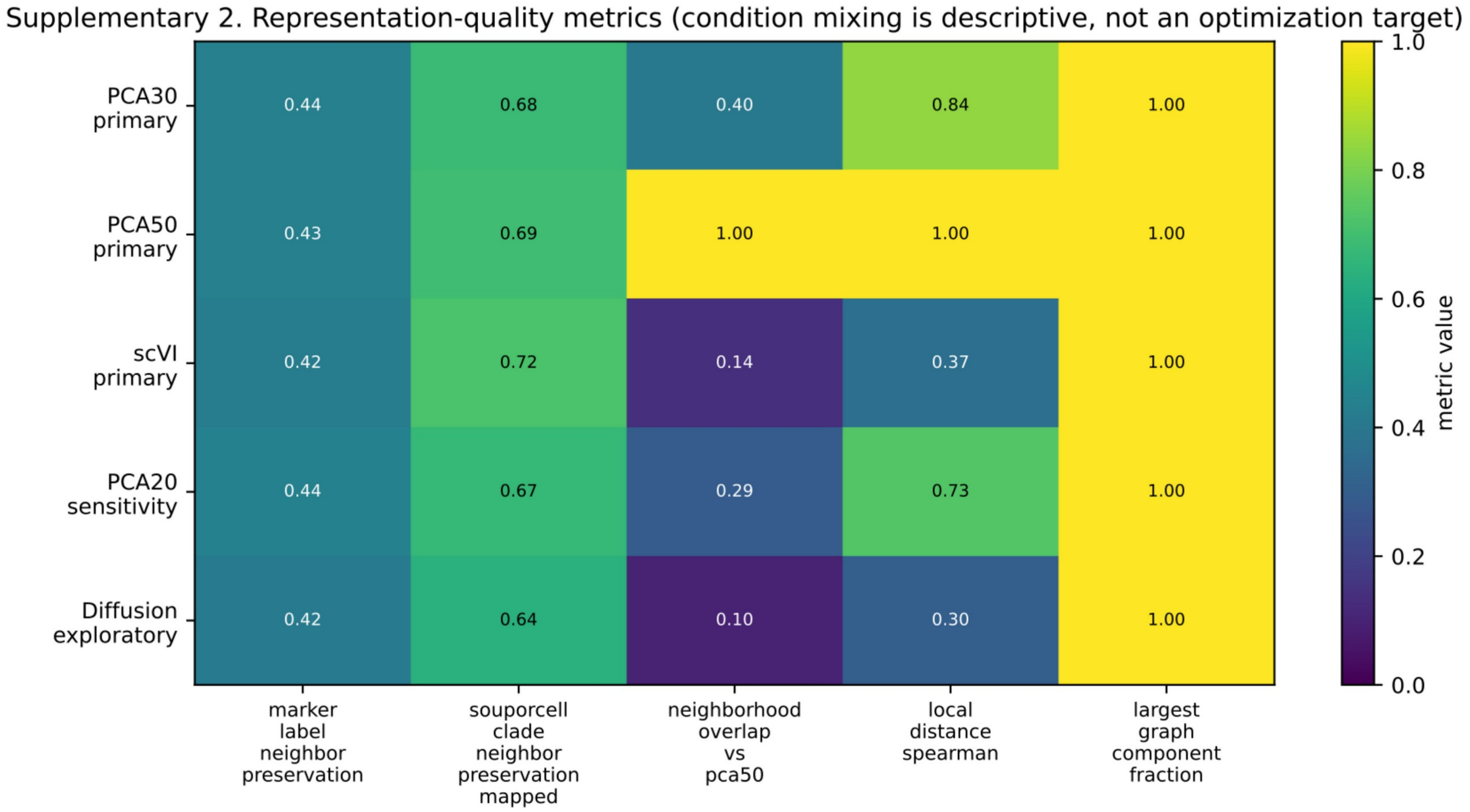

Figure S5 · band 2 of 6

Figure S5 (continued; band 3 of 6)

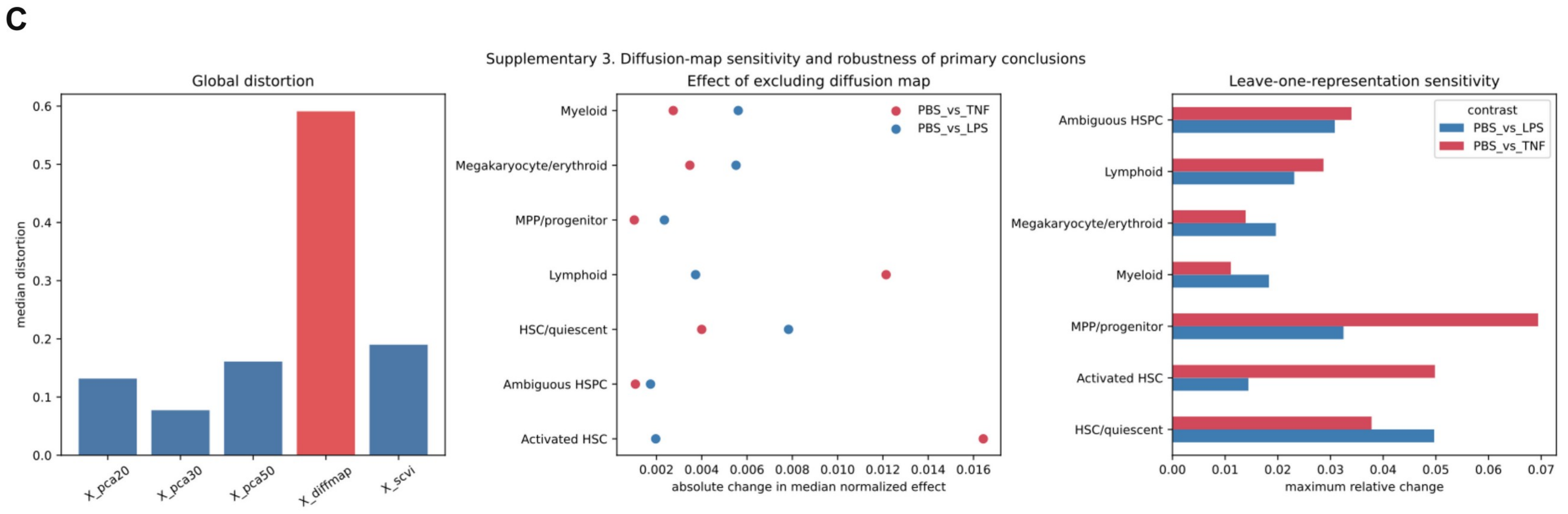

Figure S5 · band 3 of 6

Figure S5 (continued; band 4 of 6)

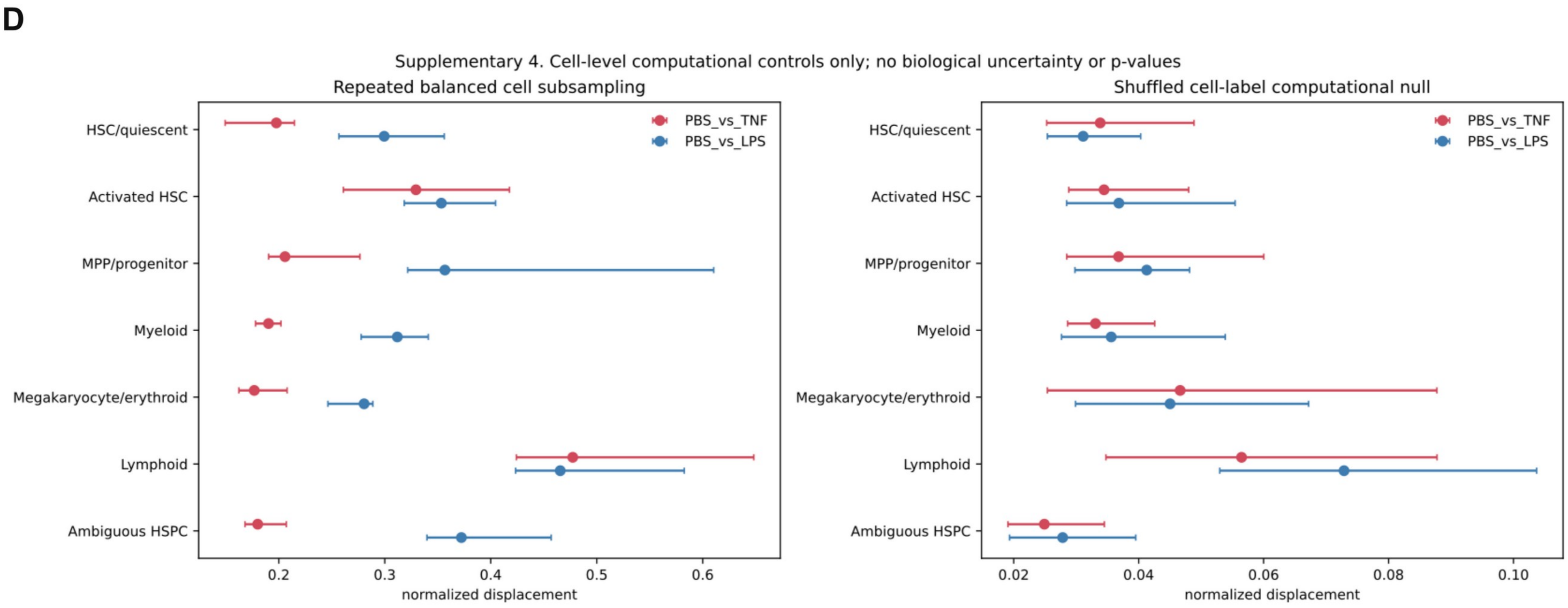

Figure S5 · band 4 of 6

Figure S5 (continued; band 5 of 6)

E

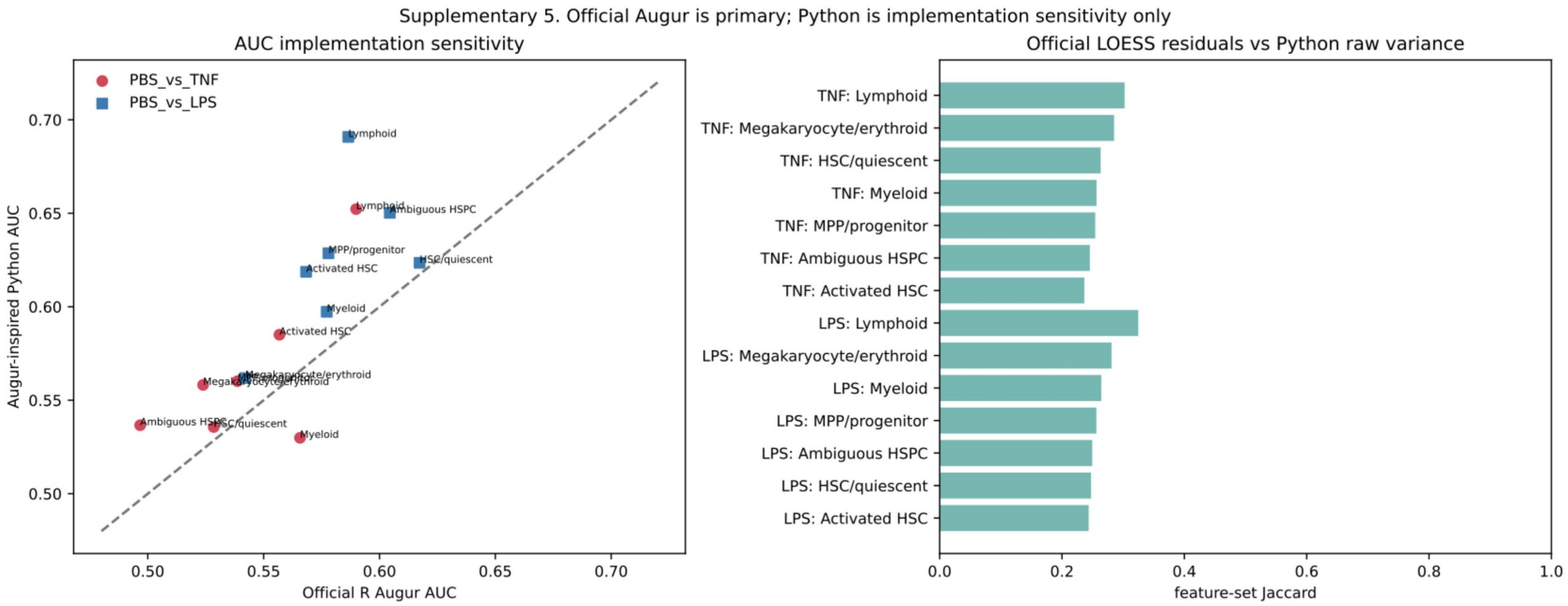

Figure S5 · band 5 of 6

Figure S5 (continued; band 6 of 6)

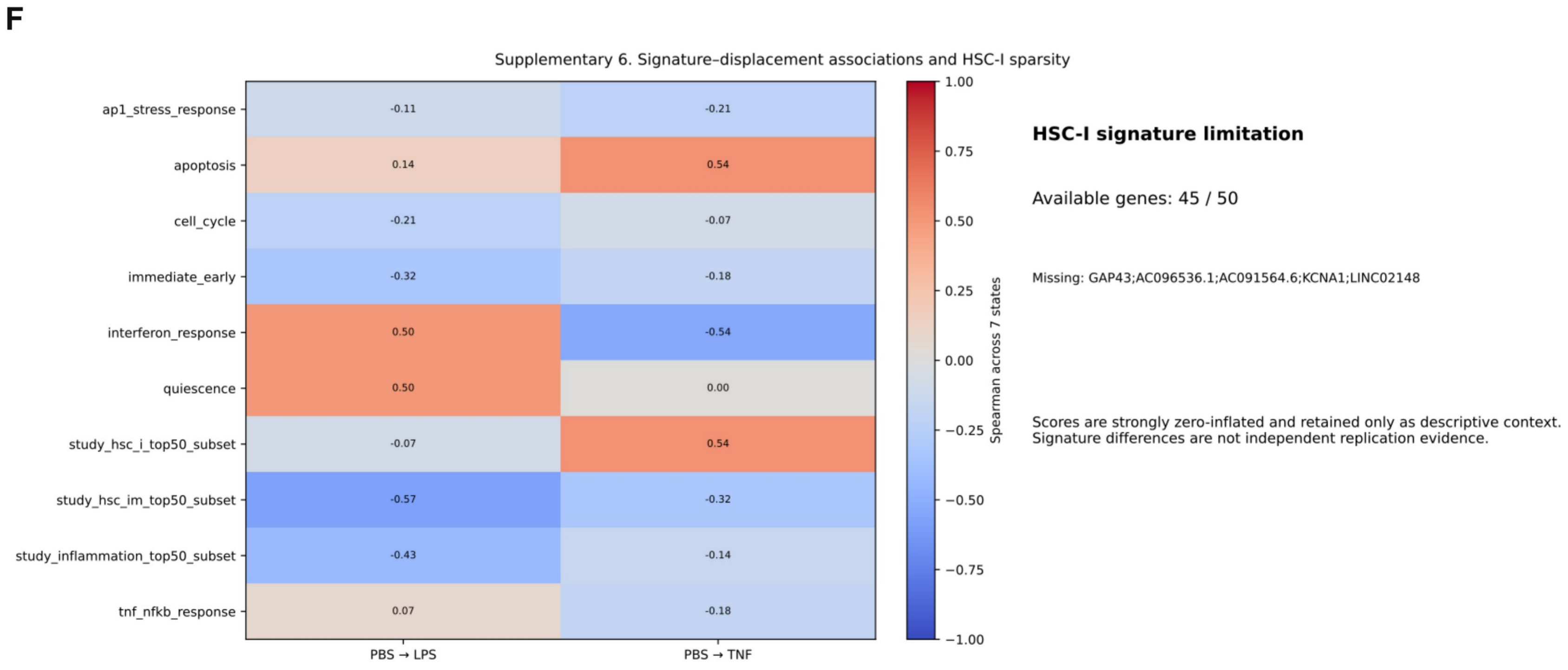

Figure S5 · band 6 of 6

Figure S6. GSE211713 replicate-aware radiation-response analysis and sample-level context

Supplementary Figure S6. Replicate-aware GSE211713 radiation-response analysis and sample-level context

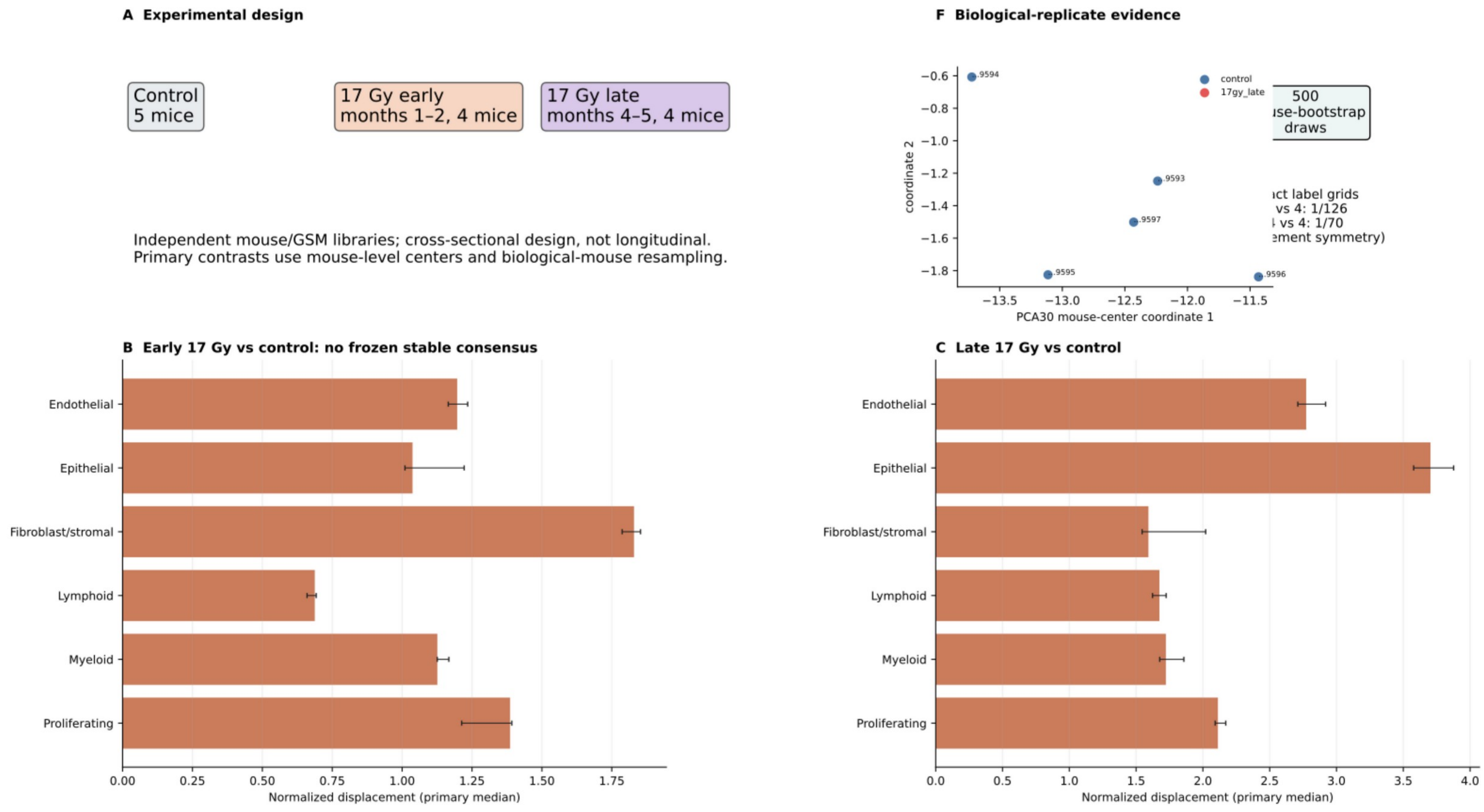

Figure S6 · band 1 of 7

Figure S6 (continued; band 2 of 7)

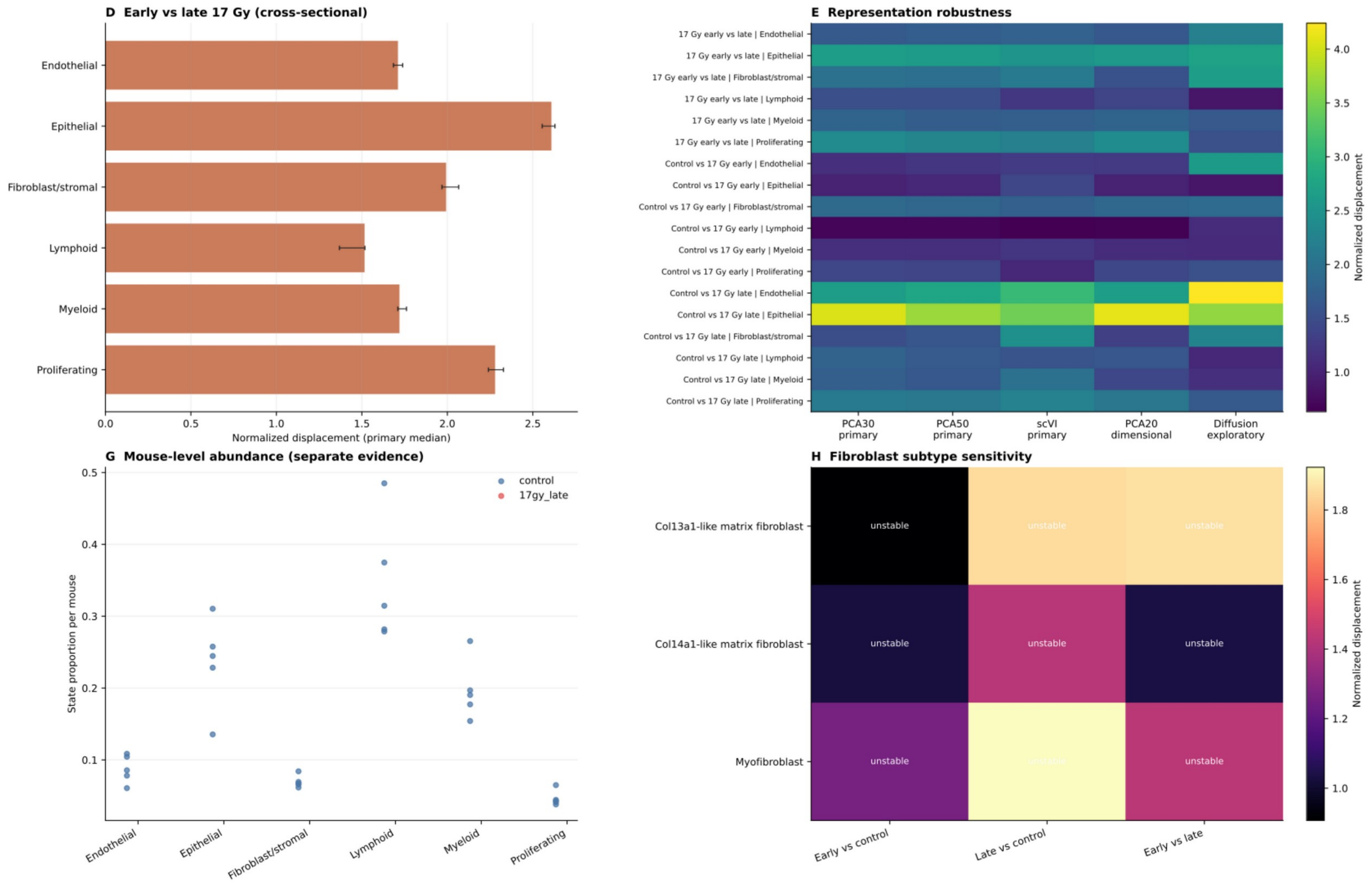

Figure S6 · band 2 of 7

Figure S6 (continued; band 3 of 7)

I

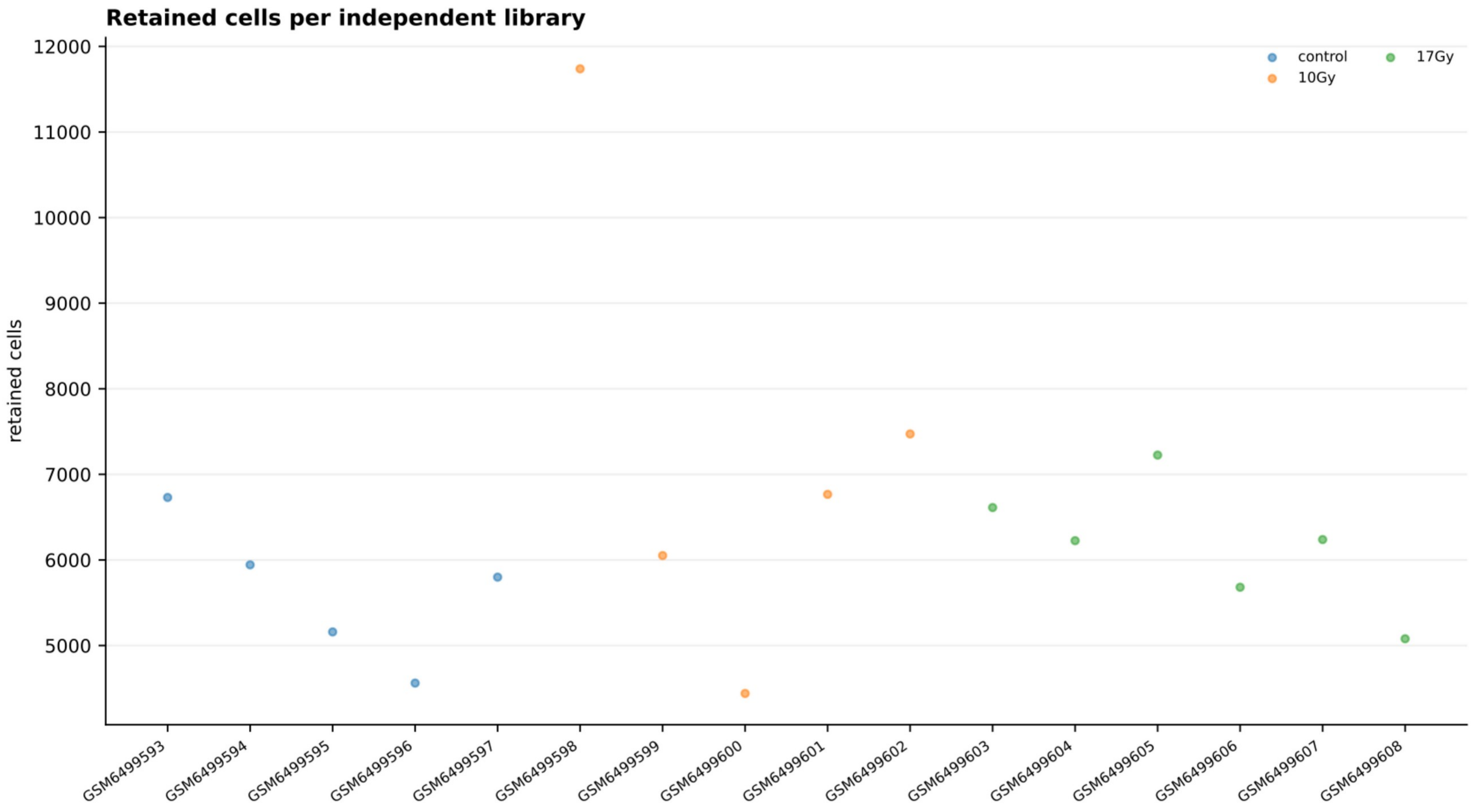

Figure S6 · band 3 of 7

Figure S6 (continued; band 4 of 7)

J

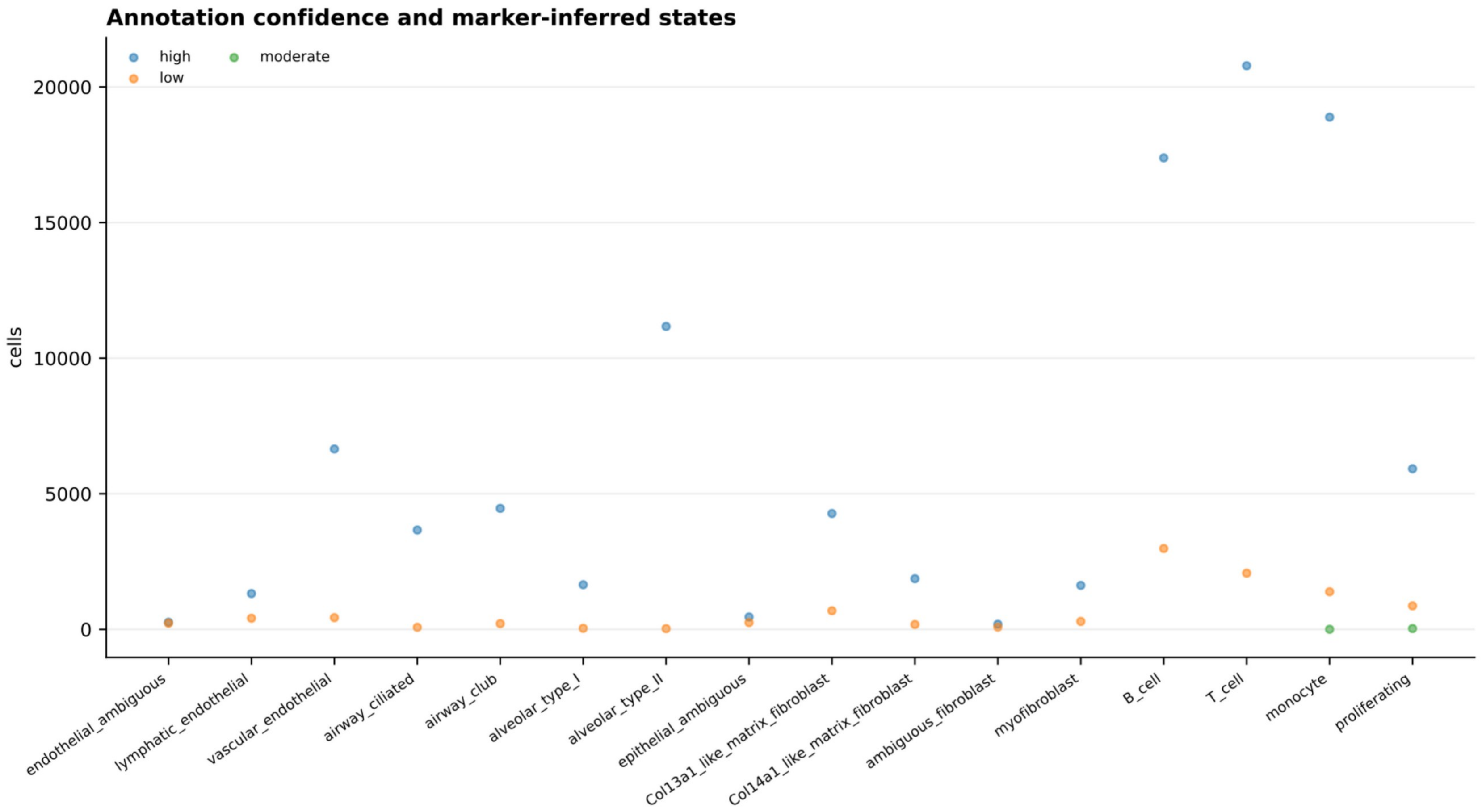

Figure S6 · band 4 of 7

Figure S6 (continued; band 5 of 7)

K

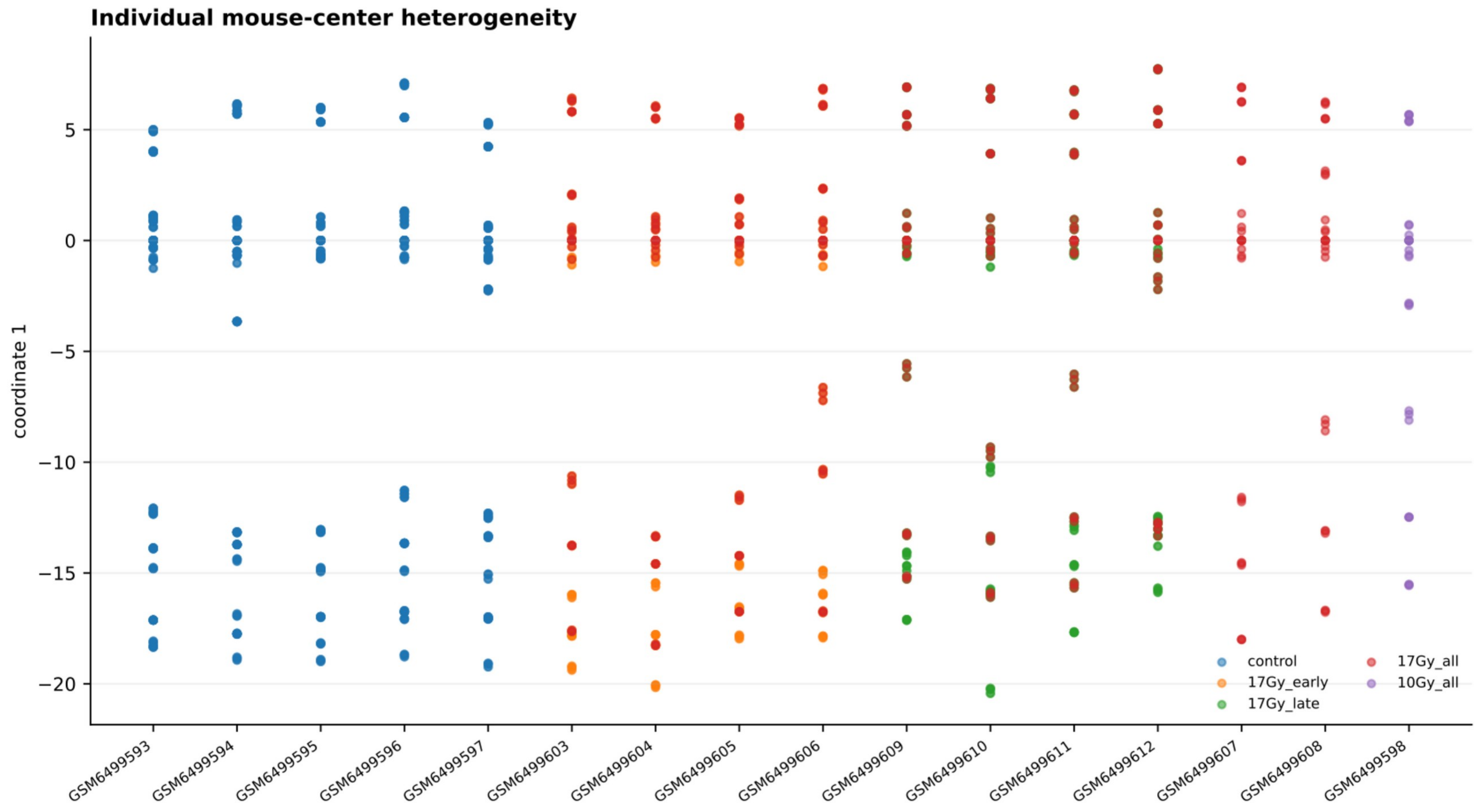

Figure S6 · band 5 of 7

Figure S6 (continued; band 6 of 7)

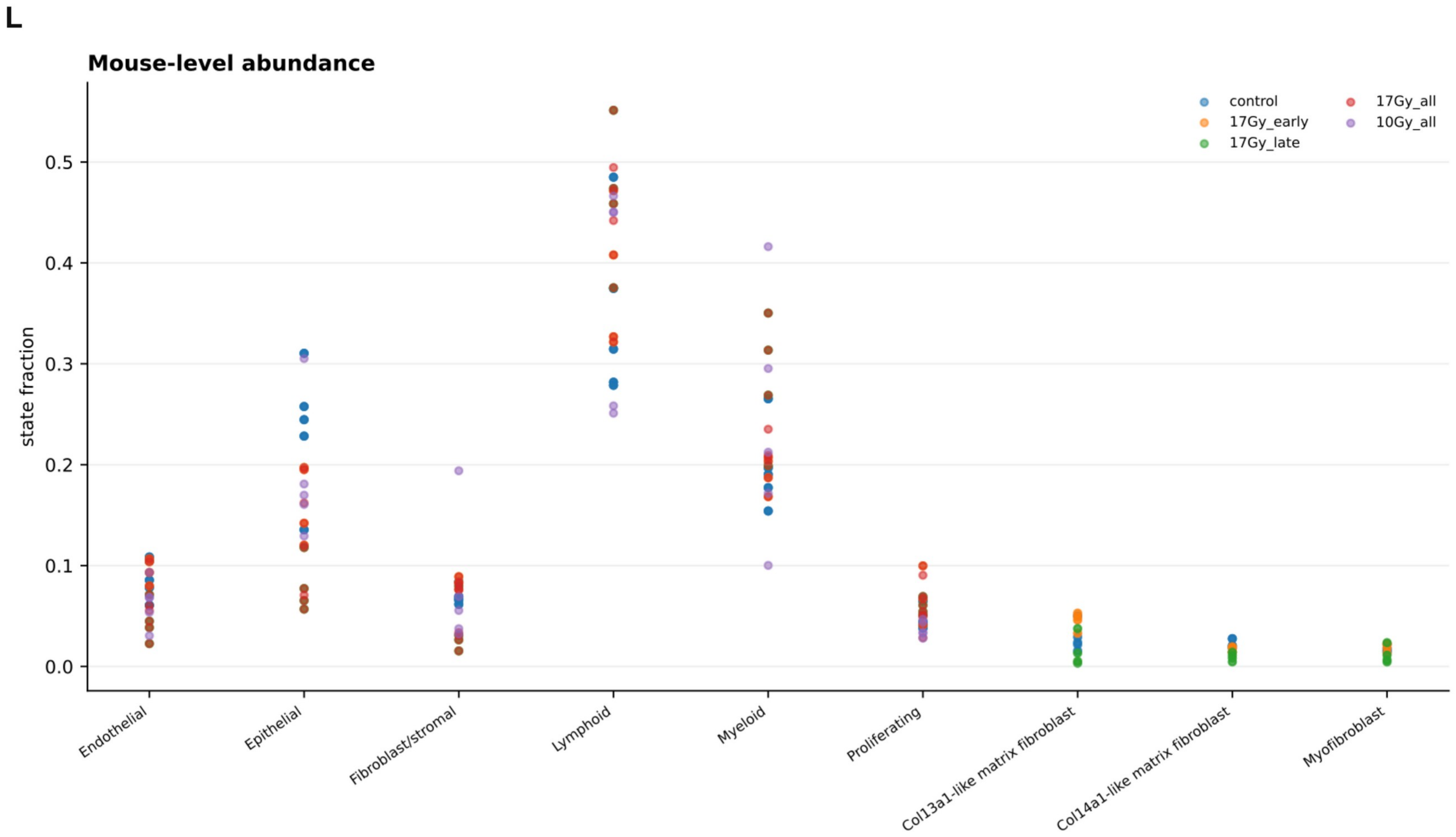

Figure S6 · band 6 of 7

Figure S6 (continued; band 7 of 7)

M

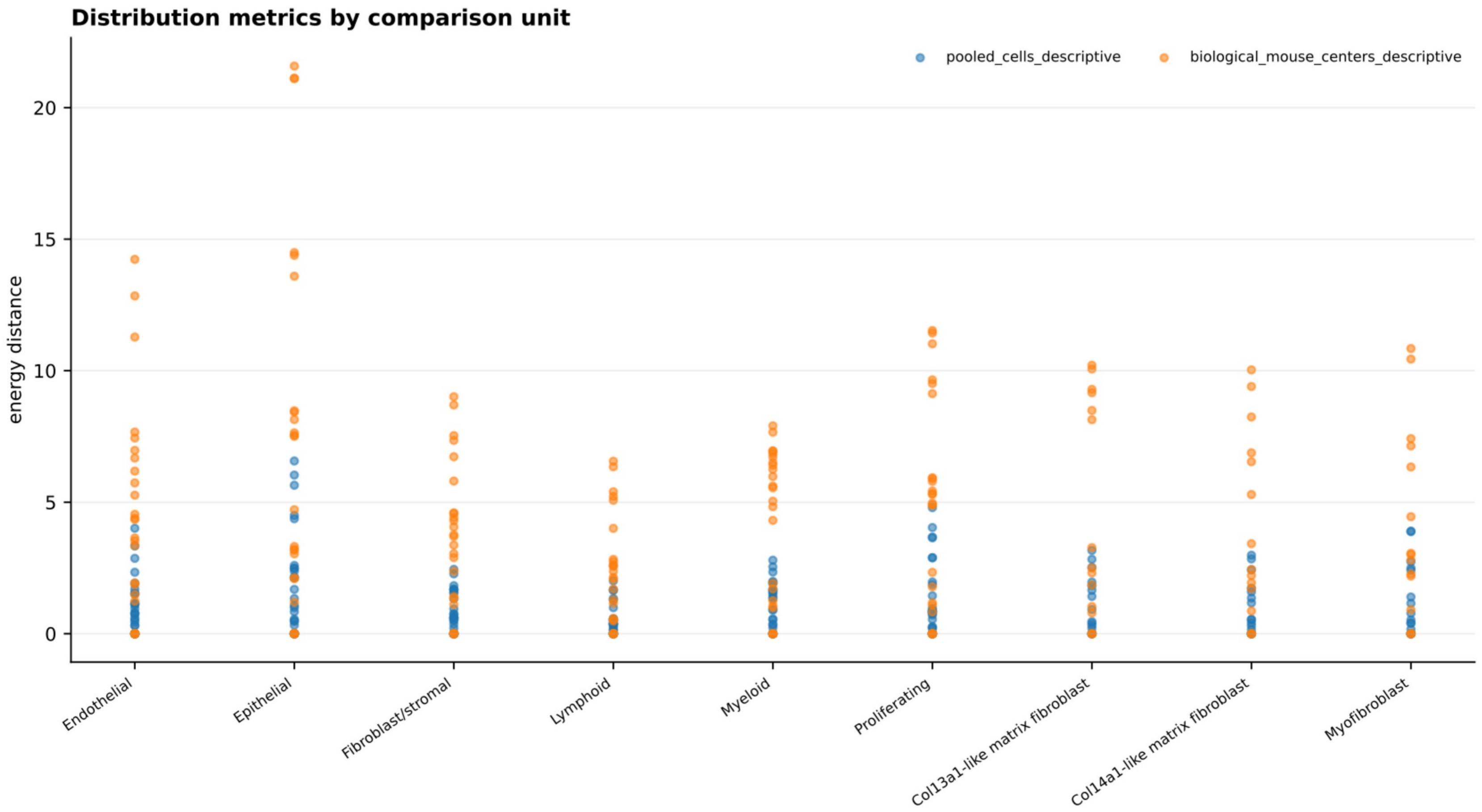

Figure S6 · band 7 of 7

Figure S7. GSE211713 mouse-level bootstrap and exact permutation inference

Supplementary Figure S7. GSE211713 biological-mouse bootstrap and exact permutation inference

A

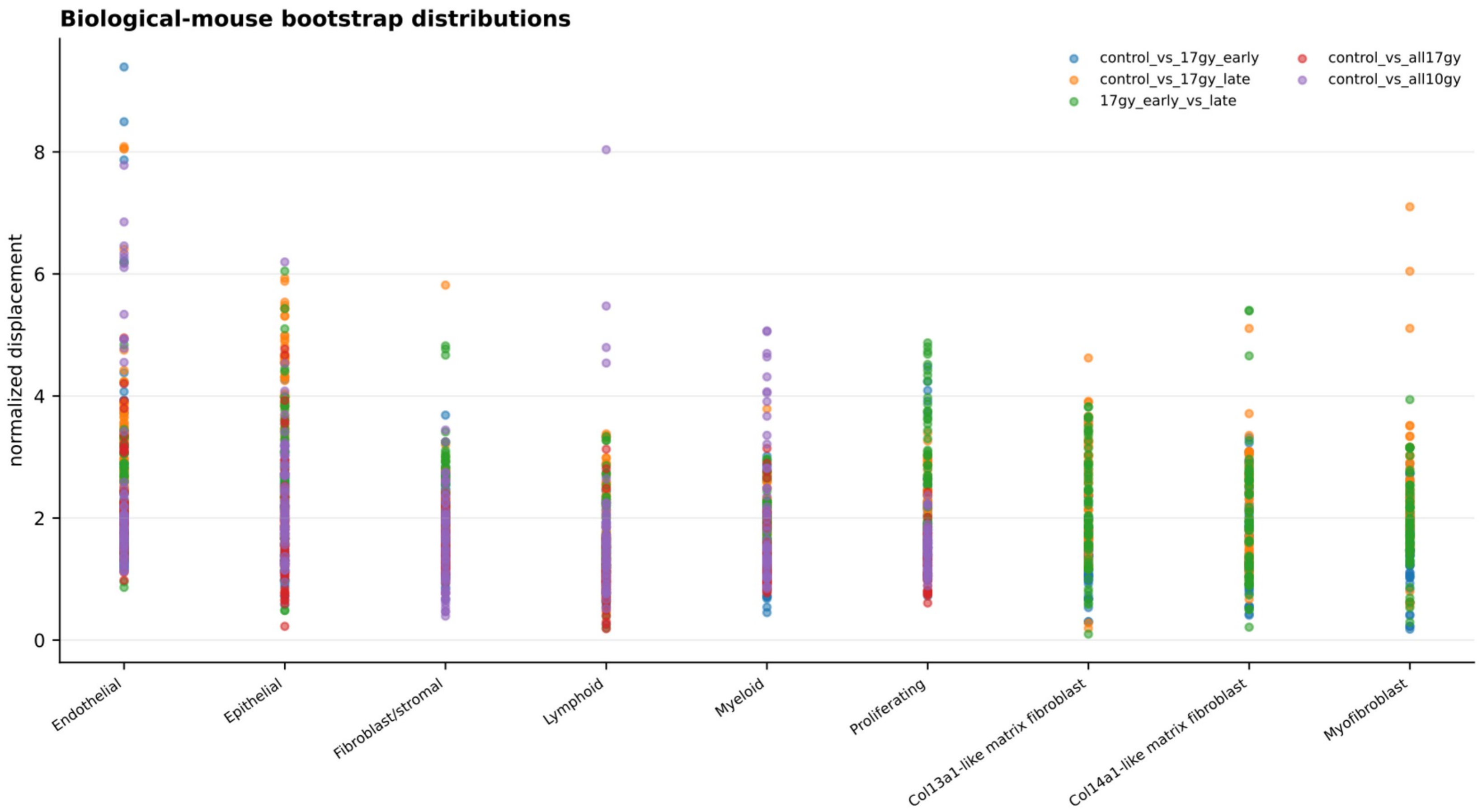

Figure S7 · band 1 of 2

Figure S7 (continued; band 2 of 2)

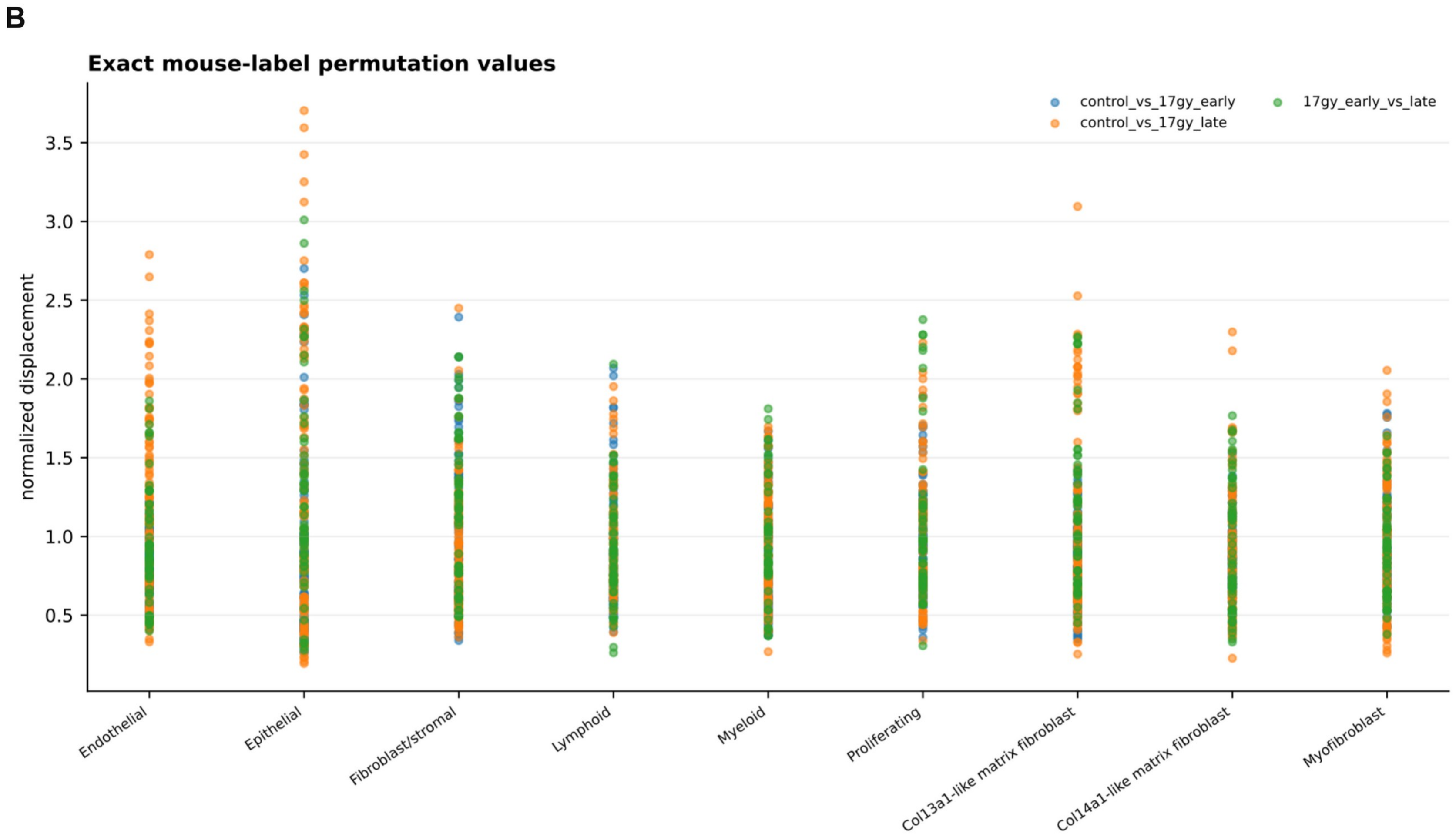

Figure S7 · band 2 of 2

Figure S8. GSE211713 representation stability, neutral findings, unstable findings, and fibroblast-subtype sensitivity

Supplementary Figure S8. GSE211713 representation sensitivity, coverage, neutral/unstable findings, and fibroblast-subtype analyses

A

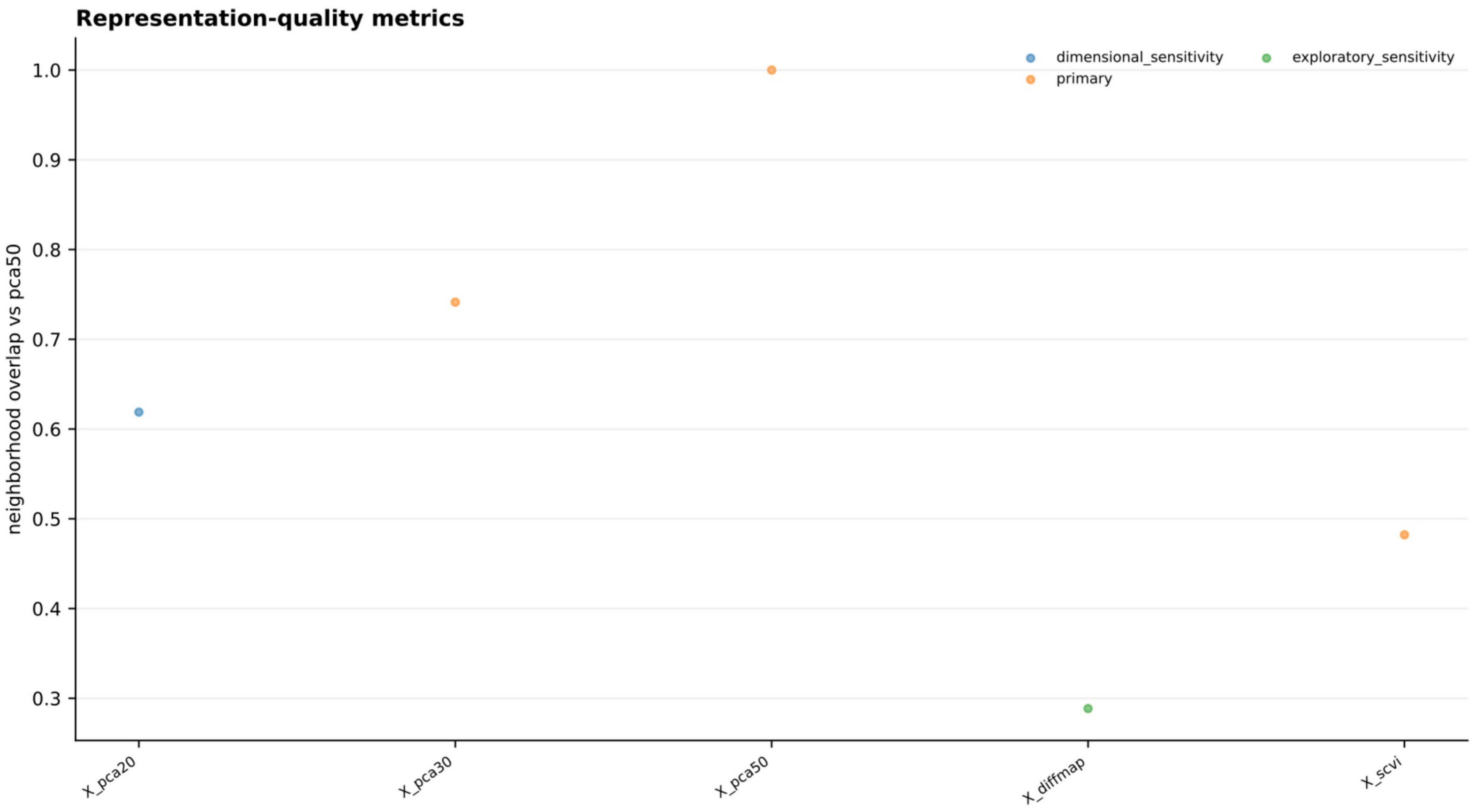

Figure S8 · band 1 of 5

Figure S8 (continued; band 2 of 5)

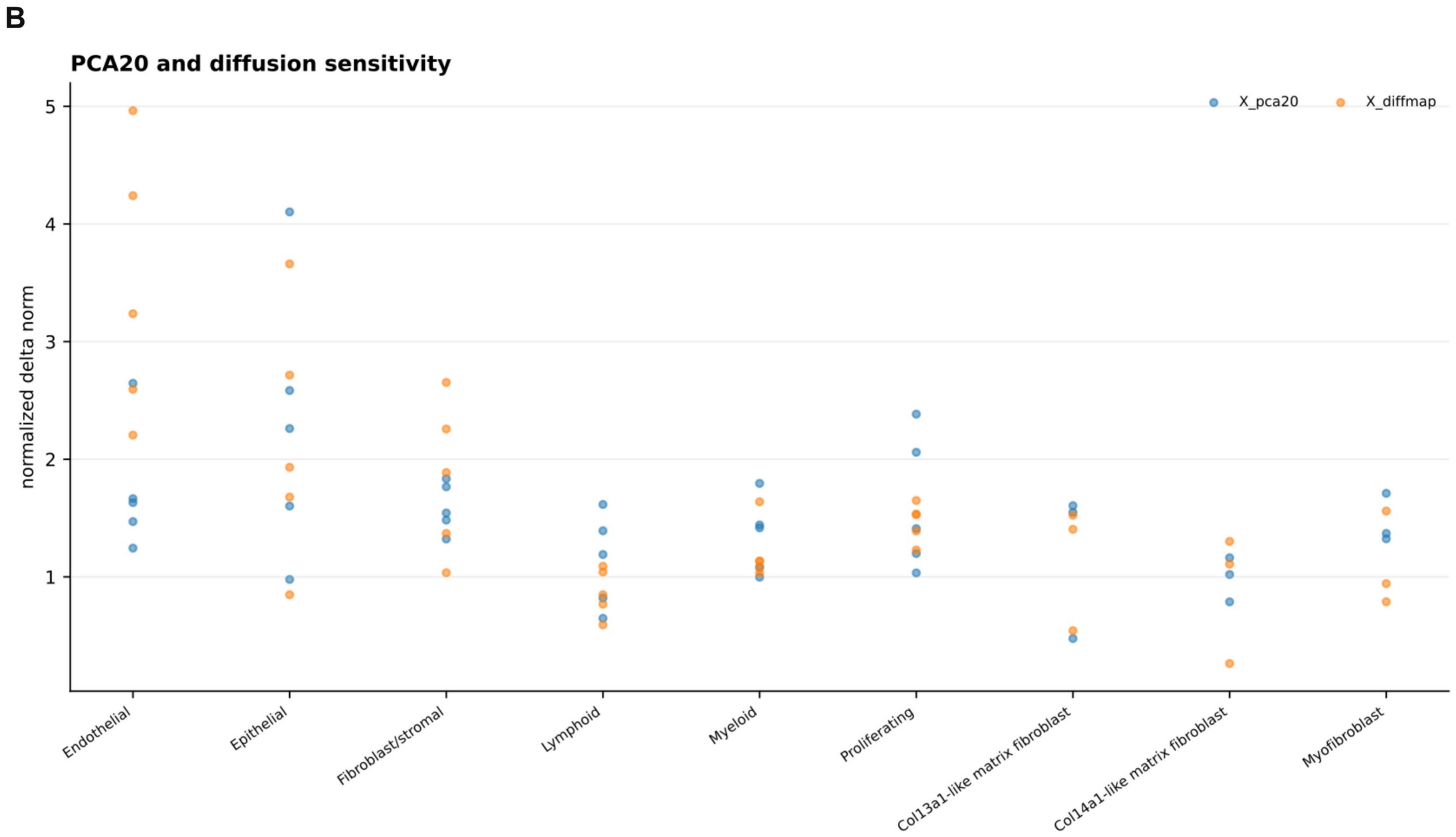

Figure S8 · band 2 of 5

Figure S8 (continued; band 3 of 5)

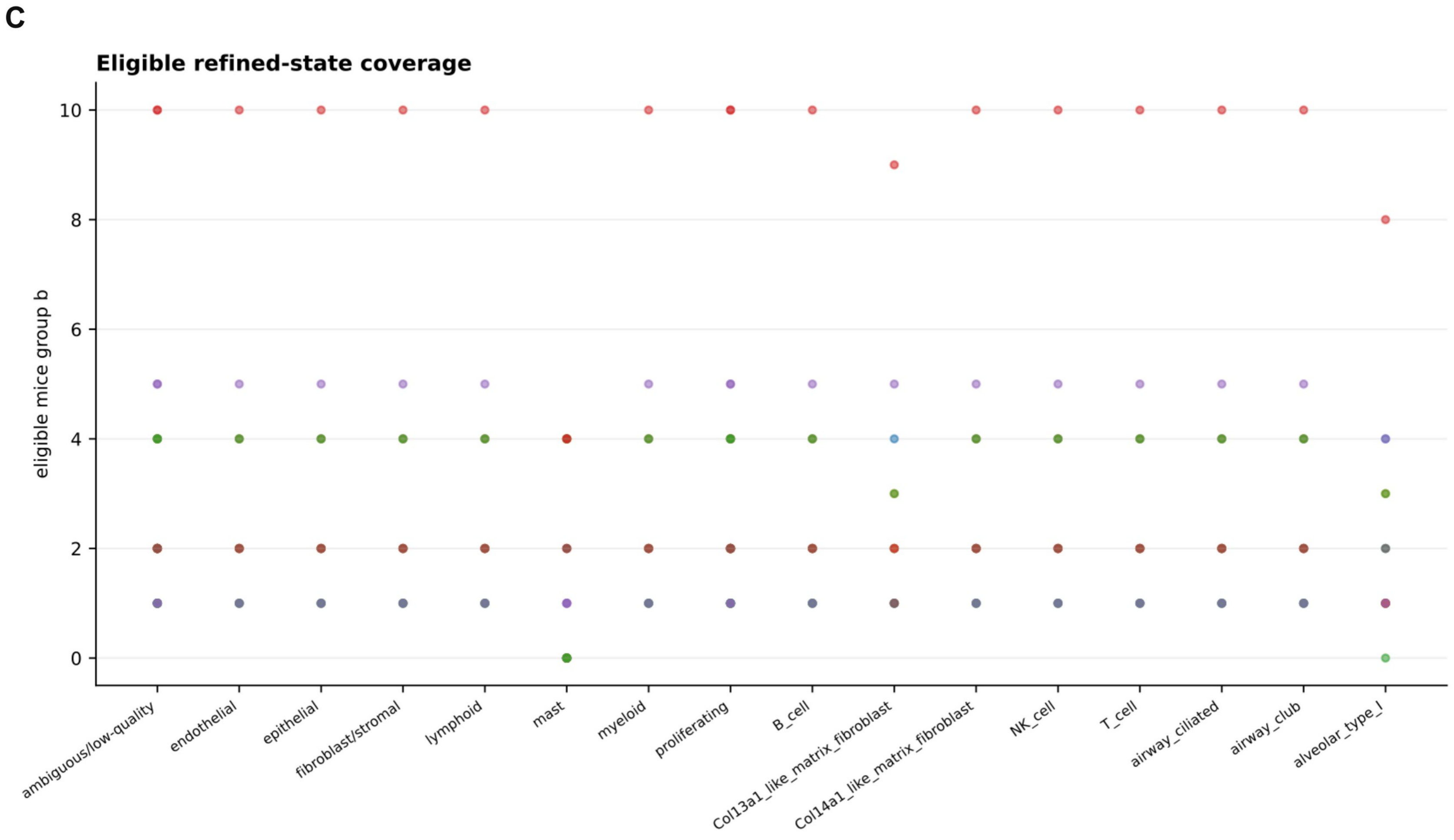

Figure S8 · band 3 of 5

Figure S8 (continued; band 4 of 5)

D

Secondary time-heterogeneous contrasts

Secondary all-17 Gy and all-10 Gy contrasts collapse heterogeneous times and are labeled replicate-aware secondary time-heterogeneous.

Figure S8 · band 4 of 5

Figure S8 (continued; band 5 of 5)

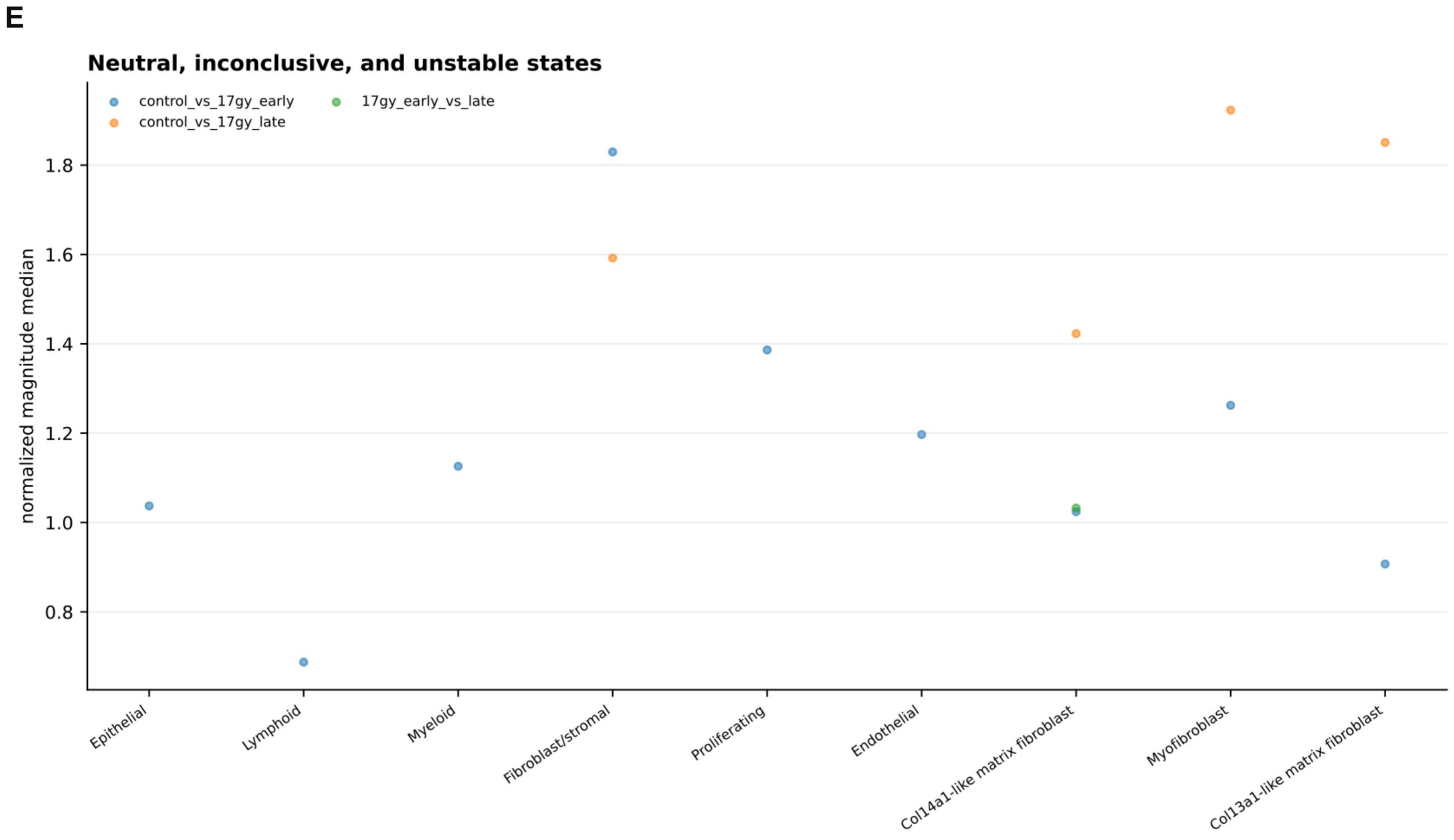

Figure S8 · band 5 of 5

Supplementary Figure Legends and Alt Text

Figure S1. Synthetic benchmark: estimator robustness and biological-sample heterogeneity

(A) Balanced simulated biological-sample heterogeneity across held-out seeds. The coordinate-wise median produced false non-neutral calls in 8 of 15 held-out jobs, the geometric median in 2 of 15, and the mean, robust mean, and trimmed mean in 0 of 15.

(B) Bootstrap assessed-state coverage by independent job with calibration and held-out evaluation separated. Held-out mean coverage was 0.805 across 105 assessed state/job combinations.

(C) Outlier-contamination displacement-magnitude error by estimator. The held-out trimmed-mean error was lowest at 0.224 in this scenario; classification was otherwise saturated.

Inference scope. Controlled synthetic evaluations; no biological claim.

Source data. Supplementary Table S2.

Alt text

Three controlled benchmark panels compare center estimators under biological-sample heterogeneity and outlier contamination. The coordinate-wise median has false non-neutral calls in 8 of 15 held-out jobs, the geometric median in 2, and three other estimators in none. Mean held-out assessed-state bootstrap coverage is 0.805. Held-out outlier-contamination magnitude error is lowest for the trimmed mean. No estimator or bootstrap profile is presented as uniformly reliable.

Figure S2. Synthetic benchmark: representation corruption, local distortion, and geometry-dynamics stress tests

(A) Held-out state-level representation-instability recall was 0.767 for the evaluated consensus, local-geometry, and dynamics variants.

(B) Fine-grained local-distortion recall was 0 in held-out representation-corruption jobs, an retained negative result.

(C) Framework-ablation success rates across failure modes, with calibration and held-out evaluation separated.

(D) Corresponding failure fractions; representation-instability and local-distortion failures remain visible.

(E) Held-out geometry stress test comparing reference, rotated-equivalent, anisotropic diagnostic, and explicitly corrupted representations. Global corruption is detected, whereas state-local distortion is not reliably localized.

(F) Consensus dynamics accuracy for aligned and discordant controlled classes; unavailable cases are excluded from success counts.

(G) Matched aligned and discordant dynamics stress tests showing control-to-treated displacement and regenerated velocity directions. Neutral-state caveats remain visible.

Inference scope. Controlled synthetic sanity checks; no biological validation.

Source data. Supplementary Table S2.

Alt text

Seven synthetic benchmark panels evaluate representation and dynamics behavior. State-instability recall is incomplete at 0.767 and local-distortion recall is zero. Ablation summaries retain failure modes. A geometry stress test detects global corruption but not reliable state-local localization. Controlled aligned and discordant dynamics classes are classified correctly when applicable. These are method tests, not biological evidence.

Figure S3. GSE132188-derived pancreatic-development geometry-dynamics validation

(A) Public pancreas UMAP with original cluster annotations and scVelo dynamical RNA-velocity flow; UMAP is a developmental visualization.

(B) Prespecified annotation-supported endocrine transition graph from Ngn3-low progenitors through Ngn3-high and Fev-positive precursors toward terminal endocrine clusters.

(C) Source-state velocity direction versus annotation-supported transition direction across PCA20, PCA30, PCA50, and diffusion representations; negative, neutral, unstable, and unavailable values are retained.

(D) Forward transitions compared with reversed-edge, shuffled-velocity, and rotated-velocity controls under the predefined cosine thresholds.

(E) CellRank GPCCA fate probabilities summarized by original cluster. CellRank uses a VelocityKernel derived from scVelo RNA velocity and is complementary, not independent.

(F) Transition-level evidence table with representation consensus, agreement fraction, comparator status, and limitations.

Inference scope. Descriptive workflow; related velocity-derived outputs are not independent experimental ground truth.

Source data. Supplementary Table S3.

Alt text

Six panels summarize the public pancreatic-development workflow derived from GSE132188: annotated pancreas UMAP and scVelo flow, a prespecified endocrine transition graph, alignment across PCA and diffusion representations, reversed-edge and altered-velocity controls, CellRank fate probabilities, and a transition evidence table. Negative and unavailable results are retained. CellRank and scVelo are related model-derived outputs, so the workflow is descriptive.

Figure S4. GSE249479 principal inflammatory HSPC analysis

(A) PCA50-derived display UMAPs colored by seven conservative marker-inferred states and by PBS, TNF, and LPS conditions; UMAP is not used for quantitative geometry.

(B) Primary-consensus normalized displacement for PBS-to-TNF and PBS-to-LPS. Stable descriptive TNF effects occur for Activated HSC and Lymphoid; all seven LPS states are stable.

(C) Representation sensitivity across PCA30, PCA50, and scVI primary views, PCA20 dimensional sensitivity, and exploratory diffusion map.

(D) Abundance change, robust displacement, and within-state energy distance shown separately with independent scales; no composite score is formed.

(E) Official R Augur 1.0.3 treatment separability versus ScGeo representation-stable displacement. The estimands are complementary, not equivalent.

(F) Inference-scope summary: no independent biological-replicate identity is available; cells, conditions, libraries, and SouporCell clades are not replicates.

Inference scope. descriptive.

Source data. Supplementary Table S4.

Alt text

Six-panel GSE249479 descriptive analysis. Display-only UMAPs show seven marker-inferred states and three conditions. Stable TNF effects occur for Activated HSC and Lymphoid, and all seven LPS effects are stable across the primary representation ensemble. Sensitivity, abundance, displacement, distributional distance, and official R Augur appear as separate quantities. No independent biological-replicate identity is available.

Figure S5. GSE249479 quality, representation sensitivity, controls, and official R Augur comparison

(A) Sparse-QC summaries and conservative marker-label confidence, including 10,154 ambiguous and 10,272 low-confidence cells.

(B) Marker-label and mapped-clade neighborhood preservation, PCA50 neighborhood overlap, local-distance agreement, and graph connectivity. Condition mixing is descriptive.

(C) Diffusion-map distortion, effect changes after excluding diffusion, and leave-one-primary-representation sensitivity. Primary conclusions use PCA30, PCA50, and scVI.

(D) Repeated balanced-cell subsampling and shuffled-cell-label controls. These quantify computational stability only and provide neither biological uncertainty nor inferential p-values.

(E) Official R Augur 1.0.3 compared with the retained Augur-inspired Python approximation. Official R output is primary; Python is implementation sensitivity, and AUCs are not averaged.

(F) Descriptive signature-displacement rank associations across seven marker-inferred states per contrast. The HSC-I score retains 45 of 50 requested genes and is strongly zero-inflated.

Inference scope. descriptive.

Source data. Supplementary Table S4.

Alt text

Six GSE249479 blocks show QC and annotation confidence, representation quality, diffusion and leave-one-representation sensitivity, computational controls, official R Augur versus a Python sensitivity implementation, and signature associations. Diffusion is more distorted than the primary views. Controls are computational, official R Augur remains primary, and all evidence is descriptive.

Figure S6. GSE211713 replicate-aware radiation-response analysis and sample-level context

(A) Cross-sectional design with five independent controls, four early 17 Gy mice at months 1–2, and four late 17 Gy mice at months 4–5.

(B) No major state met the predefined representation-consensus criteria for early 17 Gy versus control; all six were representation-sensitive.

(C) Epithelial, Endothelial, Proliferating, Myeloid, and Lymphoid states were stable for late 17 Gy versus control; Fibroblast/stromal remained unstable.

(D) All six major states met the stable consensus in the cross-sectional early-versus-late 17 Gy comparison.

(E) Primary consensus used PCA30, PCA50, and scVI; PCA20 and diffusion were dimensional and exploratory sensitivities.

(F) Individual geometric-median mouse centers, 500 biological-mouse bootstrap draws, and exhaustive mouse-label permutations expose sample-level evidence and discrete resolution.

(G) Mouse-level abundance shown separately from displacement.

(H) Fibroblast subtypes were more representation-sensitive than the major-state analysis.

(I) QC and independent-library design for all 20 mouse/GSM libraries.

(J) Conservative marker-inferred annotation counts and confidence.

(K) Individual geometric-median mouse-center heterogeneity; coordinates are representation-specific.

(L) Individual-mouse state proportions, kept separate from displacement.

(M) Pooled-cell and biological-mouse-center energy distances with comparison units exposed; these distances are descriptive.

Inference scope. Replicate-aware cross-sectional association; not longitudinal or causal. Related contrasts are not independent.

Source data. Supplementary Tables S5 and S6.

Alt text

Thirteen panels summarize the GSE211713 mouse-lung radiation analysis. Early control-versus-17 Gy effects are representation-sensitive in all six major states; late effects are stable in five states with Fibroblast/stromal unstable; all six early-versus-late cross-sectional differences are stable. Supporting panels show 20 independent mouse libraries, annotation confidence, mouse centers, mouse proportions, and descriptive distribution distances. The analysis is replicate-aware and cross-sectional, not longitudinal or causal.

Figure S7. GSE211713 mouse-level bootstrap and exact permutation inference

(A) Stored normalized-displacement draws from 500 biological-mouse bootstrap iterations per eligible state and representation.

(B) Exhaustive mouse-label permutation values with the observed assignment retained and ties counted greater than or equal to the observed statistic. Within-contrast/representation BH values are sensitivity only. The primary designs contain 126, 126, and 70 assignments.

Inference scope. Replicate-aware cross-sectional uncertainty and exact small-sample sensitivity.

Source data. Supplementary Table S5.

Alt text

Two GSE211713 panels show biological-mouse uncertainty: 500 mouse-level bootstrap draws per eligible state and representation, and every exact mouse-label assignment including the observed assignment. Ties count in the upper tail. Assignment spaces of 126, 126, and 70 yield coarse exact p-value grids.

Figure S8. GSE211713 representation stability, neutral findings, unstable findings, and fibroblast-subtype sensitivity

(A) Representation-quality diagnostics, including neighborhood overlap with PCA50; dose and time silhouettes are descriptive diagnostics rather than correction objectives.

(B) PCA20 dimensional and diffusion exploratory sensitivity, separate from the PCA30/PCA50/scVI primary ensemble.

(C) Refined-state eligibility and coverage qualification; no new numerical ScGeo analysis is introduced.

(D) Secondary all-17 Gy and all-10 Gy contrasts, explicitly labeled replicate-aware secondary and time-heterogeneous.

(E) Neutral, inconclusive, and representation-sensitive findings, including greater representation sensitivity among fibroblast subtypes.

Interpretation. Replicate-aware sensitivity analysis including negative and representation-sensitive findings.

Source data. Supplementary Table S6.

Alt text

Five GSE211713 blocks show representation-quality diagnostics, PCA20 and diffusion sensitivity, refined-state coverage, secondary time-heterogeneous contrasts, and neutral or representation-sensitive findings. Dose and time are not correction objectives. Fibroblast subtypes show greater representation sensitivity, and uncertainty is retained rather than converted into a universal stability claim.

Supplementary Table Legends

Supplementary Tables S1–S7 are provided in the accompanying workbook, with one worksheet per table.

Table S1. Datasets, designs, analysis units, and inference scope

Catalogs the controlled benchmark and four public-data workflows, their biological systems, analysis units, quantitative representations, inference classes, and principal limitations.

Table S2. Synthetic benchmark estimates and retained failure modes

Summarizes synthetic benchmark estimates from calibration and held-out evaluation, including estimator-specific false calls and magnitude error, uncertainty coverage, representation and local-distortion recall, dynamics accuracy, and ablation results.

Table S3. GSE132188-derived pancreatic transition evidence

Reports transition-level evidence for the GSE132188-derived pancreatic-development workflow, including representation agreement, velocity-derived comparator status, negative-control specificity, and visible limitations.

Table S4. GSE249479 descriptive state evidence and representation sensitivity

Reports GSE249479 descriptive state evidence for PBS-to-TNF and PBS-to-LPS, with ScGeo displacement, representation consensus, official R Augur AUC, abundance, distributional distances, and all primary/sensitivity representation values.

Table S5. GSE211713 major-state primary evidence and mouse-level uncertainty

Reports GSE211713 major-state primary-consensus evidence, biological-mouse bootstrap interval ranges, and exact permutation resolution for the three prespecified cross-sectional contrasts.

Table S6. GSE211713 neutral, unstable, and fibroblast-subtype evidence

Summarizes neutral or representation-sensitive GSE211713 findings together with fibroblast-subtype evidence, coverage, and biological-mouse uncertainty without forcing a stable-effect conclusion.

Table S7. Estimands, parameters, software, and reproducibility

Summarizes the estimands, interpretation, thresholds, group sizes, random seeds, and software settings used in the supplementary analyses.

Data and code availability

The ScGeo software and the analysis workflows used in this study are available at https://github.com/liuifrec/scgeo and https://github.com/liuifrec/scgeo-notebooks, respectively. Public datasets were obtained from GEO under accessions GSE280305, GSE132188, GSE249479, and GSE211713.

Supplementary Figures S1-S8 present the controlled simulations and public-data validation analyses, including positive, negative, and representation-sensitive findings. GSE249479 is interpreted descriptively because no independent biological-replicate identifier was recoverable. GSE211713 uses independent mouse libraries as biological samples but supports cross-sectional rather than longitudinal or causal inference.

Source data for Supplementary Tables S1-S7 are provided in the accompanying workbook, with one worksheet per table.
